# *Ab* *Initio* Exploration of the Phosphoryl Transfer Reaction Provides Insights into Interpreting Models of S_N_2 Mechanisms

**DOI:** 10.1021/acs.jpcc.5c00499

**Published:** 2025-05-14

**Authors:** Robert F. Spaine, Fei Wang, Kenneth W. Foreman, Lee A. Solomon

**Affiliations:** Department of Chemistry and Biochemistry, 3298 George Mason University, 4400 University Drive, Fairfax, Virginia 22030, United States

## Abstract

Phosphoryl transfer from nucleoside triphosphates (NTPs), which drives many chemical processes in living organisms, is a putatively concerted (S_N_2) process. However, some computational studies have found dissociative (S_N_1) character. In this work, we model the hydrolysis of the terminal phosphoryl group of a magnesium-bound methyl triphosphate using the ωB97X-D4//6-311++G­(d,p) level of theory. We recapitulate experimental activation barriers for aqueous hydrolysis. We also vary solvent conditions from an explicit water shell in implicit water to implicit acetone and to implicit water with lowered dielectric equal to that of acetone. In all environmental conditions, we observed a concerted chemical mechanism, yet with decreased bond orders in the transition state. Furthermore, these bond orders decrease with increasing electrostatic repulsion and possibly steric repulsion, suggesting that diffuse interactions dominate the transition state and lead to underestimated bond orders. To quantitate the concerted aspect, we combine fractional progress in bond length and bond order for the transformation from the reactants to the products at the transition state. We find that both the attacking nucleophile and leaving group have similar sums of fractional progress. Additionally, enzymatic phosphoryl transfer likely benefits from favorable steric interactions and the lower dielectric expected in protein pockets compared to water

## Graphical abstract

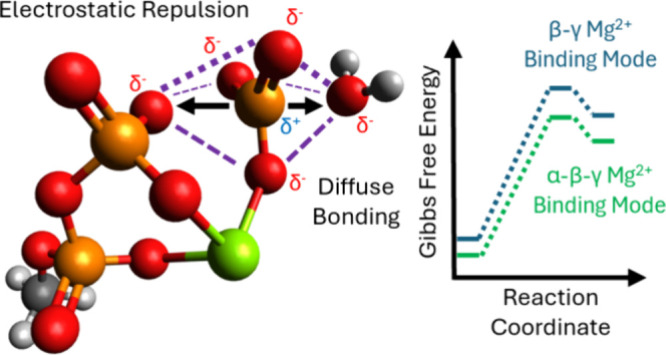

## Introduction

S_N_2 reactions involve nucleophilic substitutions in which the reaction rate is first order in each of the nucleophile and substrate. By implication, both species are present in the rate-limiting step. This step corresponds to a single transition state with no intermediates forming between reactant and product states. Of necessity, while the bond to the leaving group within the substrate starts to break, a bond with the nucleophile starts to form. Despite these broad-stroke commonalities, S_N_2 reactions show significant variability in their behavior as a function of the nucleophile basicity, substrate steric interactions, and surrounding solvent conditions.

Most experiments to probe these effects employ kinetic isotope effects (KIEs) on or linear free energy relationships (LFERs) among reaction rates.^1–3^ A broad group of S_N_2 reactions appears as “loose”, meaning that significant bond breaking between the leaving group and the rest of the substrate occurs at the transition state. Information on the strength of the nucleophilic bond at the transition state is more limited but suggests they may be weakly formed.^2^ These experimental results create a conundrum in that the bonds, largely lacking in the S_N_2 transition state, still manage to hold the complex together so no intermediates form.

In response, a large number of computation studies have considered various aspects of the mechanism. Importantly, although gas phase studies carry many of the aspects of S_N_2 reactions, the energy barriers and geometrical conformations at the transition state are often inconsistent with the experiment. Solvated models appear essential.^4^ Generally, S_N_2 reactions favor backside attack, with inversion of configuration in the product, although a front-side attack with conservation of configuration is possible in special cases.^5^ Measures of looseness in the transition state focus either on the percentage increase and decrease in bond lengths or on the differential total bond orders between the central atom and both the attaching nucleophile and the leaving group. Total bond orders in the transition state tend to be less than that of the substrate or product, suggesting a generic looseness to the reaction mechanism. Most agree that a near 180° angle between attacking and leaving atoms through the inverting center at the transition state is a conserved feature. Nevertheless, what constitutes the signature of an S_N_2 mechanism in contrast to a dissociation + association (stepwise dissociative) mechanism remains a somewhat open quantitation question.

The S_N_2 reaction of transferring a terminal phosphoryl group to an attacking nucleophile is one of the more consequential reactions in biology. Enzymes transfer phosphoryl groups to biologically relevant substrates (e.g., kinases) or water (e.g., phosphatases). These reactions impact myriad cellular functions including energy metabolism, information transfer, homeostasis maintenance, cell replication, and active transport.^6–13^ Inhibition of this activity (e.g., through mutation) often proves fatal or severely incapacitating at the cellular level. For example, cystic fibrosis involves mutation of the ATP binding sites of the CFTR channel, resulting in several chronic pathologies.^8,14^ Developing a more complete picture of how enzymes catalyze phosphoryl transfer assists not only attempts to treat such diseases but also the creation of more efficient enzymes.

Engineered synthetic enzymes leverage phosphoryl transfer reactions in industrial settings.^15–17^ To improve the synthetic efficiency of β-nicotinamide mononucleotide, a compound of interest in antiaging research, the catalytic site of human nicotinamide riboside kinase was engineered to improve ATP-dependent phosphoryl transfer.^16,17^ Rational engineering of kinase function relies on a first-principles understanding of how the chemical properties of catalytic protein groups lower the activation barrier of phosphoryl transfer.^8,16–19^ While certain groups have designed phosphoryl transferase sites, the influence of individual amino acids from the active site on the mechanistic pathway and therefore the transferable principles behind their catalytic activity remain unclear.^15–20^ Given the complexities of protein-mediated catalysis, computational efforts have focused on simpler model systems to capture the critical characteristics of the underlying process including methyl triphosphate or adenosine triphosphate in aqueous solvent.^21–27^ These model systems have led to deeper insights into the underlying mechanism itself.

QM cluster and QM/MM approaches have been applied to enzymatic phosphoryl transfer processes.^28–31^ QM cluster studies have described how specific protein environments and leaving groups affect transition state structure in phosphoryl transfer processes.^28,29^ The QM/MM approach has also provided insights into the mechanistic pathways of phosphoryl transfer in specific protein environments.^30,31^ However, precisely controlling implicit solvent properties, permits understanding of the fundamental physical properties affecting the mechanism and activation barrier of phosphoryl transfer.

Phosphoryl transfer reactions are modeled as resting on a two-dimensional reaction plane, classified between three classical mechanistic pathways: concerted, stepwise associative, and stepwise dissociative (Scheme 1).^1,32,33^ In the discussion of these mechanistic scenarios, an intermediate refers to an observable energetically metastable state, a structure at a local energetic minimum in the reaction pathway.^1^ The concerted mechanism proceeds through an S_N_2-type transition state with simultaneous bond breaking and formation of equal and opposing magnitude. The associative (association-decomposition) mechanism begins with a nucleophilic attack directed at the phosphorus center of the terminal phosphoryl group without displacement of the leaving group. This mechanism results in a penta-coordinate phosphorane intermediate in which the phosphorus atom bonds to both the attacking nucleophile and the leaving group. The formation of a metaphosphate intermediate defines the dissociative (S_N_1-type) mechanism. In this mechanism, the terminal phosphoryl group dissociates from the substrate, forming a metaphosphate intermediate before associating with the attacking nucleophile.^1,24,25,32,33^ Dissociative mechanisms allow for potential alternate products to form and are generally termed “loose”. Some concerted reactions feature nonideal bond orders at the transition state, favoring either the reactant bond order (early, associative) or the product (late, dissociative) bond order. No *a priori* concept predicts the phosphoryl transfer mechanism, nor exactly how solvents contribute to pathway character. Further, any particular attachments to the phosphorus or changes in the local environment could place the transition state anywhere on the reaction plane.

**Scheme 1:**
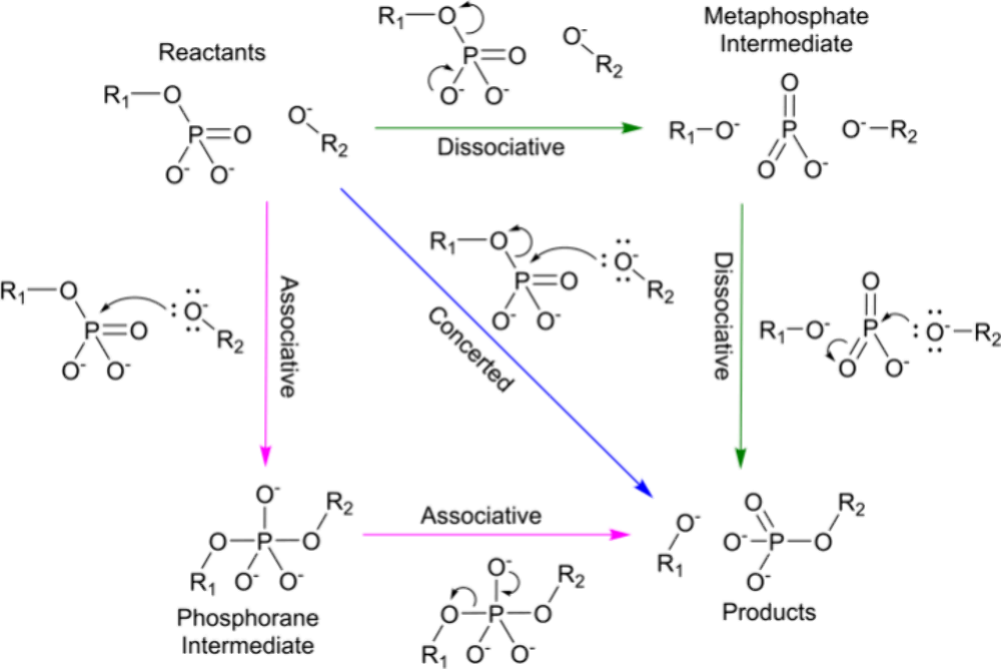
Graphical Summary of the Concerted (Blue Arrow), Stepwise Associative (Pink Arrows), and Stepwise Dissociative (Green Arrows) Pathways for Phosphoryl Transfer in which the Attacking and Leaving Atoms Are Both Oxygens

Previous computational studies disagree regarding the favored mechanism in an aqueous environment.^21–27^ Early studies did not have dispersion corrections, despite their relevance to the DFT optimization of molecular geometries. Akola and Jones used DFT-based Car–Parrinello molecular dynamics (CPMD) to characterize magnesium-methyl triphosphate (Mg·MeTP^2–^) hydrolysis reactions; they modeled the pathway either intentionally as associative or dissociative, or flexibly and concluded that a dissociative pathway is most favorable.^26^ The metadynamics work of Glaves et al. likewise concluded a dissociative or S_N_1-type process for Mg·MeTP^2–^hydrolysis.^21^ Harrison and Schulten used QM/MM to compare associative and dissociative mechanisms of magnesium-adenosine triphosphate (Mg·ATP^2–^), ultimately concluding that a dissociative mechanism is more favorable.^27^ Wang et al. performed a QM/MM study of Mg·ATP^2–^ hydrolysis using the Nudged Elastic Band (NEB) method and unlike the previously mentioned studies, their results favor a concerted pathway.^22,34^ More recent studies have a dispersion correction, but these corrections are limited in accuracy because they do not account for atomic partial charges.^35–40^ Barrozo et al. studied Mg·MeTP^2–^ hydrolysis using DFT and concluded a concerted pathway prevails.^23^ Yamabe et al. investigated Mg·ATP^2–^ hydrolysis using DFT and also concluded an S_N_2-type or concerted pathway was more likely.^24^ Finally, Saxena et al. presented a metadynamics study of Mg·ATP^2–^ hydrolysis that suggested a single-step dissociative pathway.^25^ Despite these efforts, the nature of the nucleoside triphosphate hydrolysis remains unclear, particularly concerning the extent of dissociative vs concerted character and its dependence on solvent properties. Given that QM and QM/MM approaches have predicted activation barriers with similar accuracy, a strictly QM approach likely suffices to characterize phosphoryl transfer processes.^21–27,41–43^ Yet, a more advanced level of DFT theory is necessary for accurate pathway characterization. The more recent DFT-D4 dispersion correction allows for more accurate optimization of molecular geometries and energies, particularly for charged and highly polar systems.^35,36^ Nevertheless, this uncertainty among models, coupled with the two-dimensional reaction space, generates concerns in modeling experimental outcomes under a particular set of conditions.

In this work, we performed a QM study of Mg·MeTP^2–^ hydrolysis, characterizing the mechanism within the conceptual scheme shown in Scheme 1. We add to the current understanding of Mg·MeTP^2–^ and Mg·ATP^2–^ hydrolysis mechanisms by describing the effects of solvent, electrostatics, and steric interactions on the geometry and bond orders of the transition state. The reaction pathways appear neither stepwise dissociative nor stepwise associative, but rather only concerted. We suggest that seemingly dissociative bond orders at the late transition state result from significant contributions from diffuse orbitals and electrostatic repulsion between the planar phosphoryl (PO_3_) group and the attacking and leaving oxygen atoms on either side of it. Our results also suggest possible contributions from solvent steric effects to this dissociative bond order character. We suggest a metric that may help quantitate S_N_2 reactions. Finally, our results suggest that the lower dielectric of enzymatic active sites, relative to aqueous solvent, helps decrease the activation barrier of phosphoryl transfer.

## Methods

### General Methods

All geometry optimizations, nudged elastic band (NEB) runs, vibrational frequency calculations, and population analyses in the present work were run in ORCA 6.0.0,^34,44–53^ using the ωB97X-D4 functional.^54^ We selected the 6-311++G­(d,p) basis set to model electron orbitals.^55^ The 6-311++G­(d,p) basis set has polarization functions on both heavy atoms and hydrogen atoms, improving the modeling of hydrogen-bond interactions.^55^ Our calculations used the Solvation Model based on Density (SMD) implicit solvation model with water as the solvent unless otherwise specified.^56,57^ The SMD implicit solvation model mimics the electrostatic and dispersion interactions between the explicit molecular system and the bulk solvent environment.^56,57^ The ORCA implementation of the SMD model accounts for dielectric, solvent radius, refractive indices at 293 and 298 K, and Abraham’s hydrogen bond acidity and basicity parameters, relative macroscopic surface tension, aromaticity, and electronegative halogenicity.^56,57^ Convergence thresholds for geometry optimizations and NEB runs are presented in detail in the Supporting Information.

The enthalpy, entropy, and Gibbs free energy are mostly calculated at a pressure of 1.00 atm and 298.15 K. The remaining calculations employed a temperature of 333.15 K. Bond orders were calculated using Mayer population analysis.^58–60^ Atomic partial charges were calculated using Löwdin population analysis.^61^ All molecular modeling and three-dimensional renderings of molecular structures were completed using Avogadro 1.2.0.^62,63^

Computations necessary for this research were run on the HOPPER cluster provided by the Office of Research Computing at George Mason University.^64^

### Solvent versus Substrate Assistance

We considered that proton transfer in phosphoryl transfer reactions may proceed through a solvent-assisted or substrate-assisted mechanism.^23,25,32^ In a solvent-assisted mechanism, proton transfer is mediated through a hydrogen bond network of solvent molecules.^23,25,32^ In a substrate-assisted mechanism, a nucleophilic part of the substrate directly abstracts a proton from the lytic species, allowing the completion of the nucleophilic attack.^23,25,32^ A substrate-assisted mechanism has been implied in a QM/MM study of ATP hydrolysis, but this can be interpreted as a result of applying QM theory to the lytic water molecule and classical mechanics to all other solvent molecules, artificially preventing a solvent-mediated proton shuttle.^22^ Metadynamics and DFT studies favor solvent-assisted mechanisms for Mg·ATP^2–^ and Mg·MeTP^2–^ hydrolysis reactions.^21,23,25^ Considering the structure of the attacking nucleophile, experimental evidence suggests that an increase in pH from 6.59 to 8.25 may not increase the rate of Mg·ATP hydrolysis.^65^ Therefore, we reason that the attacking nucleophile during the activation step is less likely a hydroxide ion but rather an intact water molecule and that the attack is assisted through hydrogen bonding between the lytic water and the solvent (Figure 1). P_γ_ is the phosphorus center of the γ-phosphoryl group, O_l_ is the leaving oxygen that bridges the β- and γ-phosphoryl groups in methyl triphosphate, and O_nuc_ is the nucleophilic oxygen in the water that attacks the phosphorus center of the γ-phosphoryl group. In the present work, we only characterize this activation step, even if it ends with a metastable intermediate. We do not describe the low-energy processes after the activation step. Depending on the solvent, these low-energy processes are expected to include proton shuttles and the rearrangement of noncovalent interactions.^21,23,25,66^

**Figure 1:**
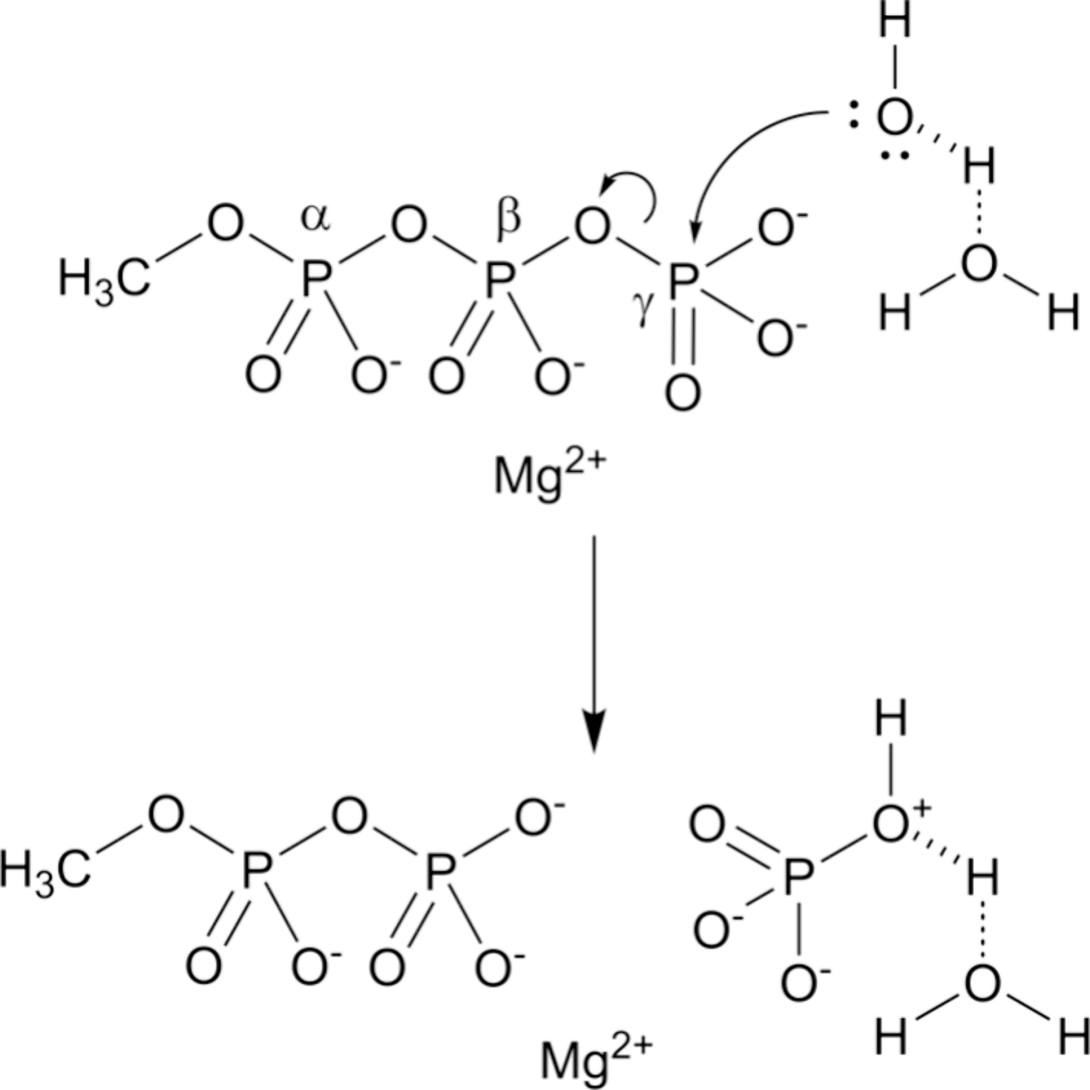
Solvent-assisted mechanism for phosphoryl transfer to water from Mg·MeTP^2–^. The phosphorus atoms of the α-, β-, and γ-phosphoryl groups are labeled in the reactant state.

### DFT Dispersion Correction

*In silico* studies of Mg·MeTP^2–^ or Mg·ATP^2–^ hydrolysis used either no dispersion correction,^21,22^ an adaptation of Grimme’s D2 (in ωB97X-D),^23,24,37,39^ or Grimme’s D3.^25,38^ We instead use the ωB97X-D4 functional, which contains a built-in DFT-D4 dispersion correction.^54^ Unlike the D2 scheme, D3 and D4 calculate C_6_ dispersion coefficients using dynamic polarizabilities computed using time-dependent density functional theory (TD-DFT), and fractional atomic coordination numbers.^36,35,38,37^ However, in D4, the scaling of polarizabilities also uses atomic partial charges.^35^ The charge dependence of D4, lacking in the D2 and D3 schemes, benefits systems having large atomic charges, such as polar and ionic systems.^35–38,40,54^ This additional charge dependence makes the D4 scheme particularly applicable to calculations on Mg·MeTP^2–^ which involves charged species and numerous dipoles throughout the system.^35,36,54^ Therefore, we leveraged this theoretical advance to characterize the reaction pathway of Mg·MeTP^2–^ hydrolysis more rigorously than in previous *in silico* studies.

### Optimization of Energetic Minima

The optimization of reactant and product states began with atomic coordinates from Barrozo et al.^23^ The coordinates corresponded to the solvent-assisted hydrolysis of Mg·MeTP^2–^ with magnesium bound in “mode 3” (to the α-, β-, and γ-phosphoryl groups of MeTP^4–^) or “mode 1” (to the β- and γ-phosphoryl groups of MeTP^4–^).^23^ These structures result from direct optimization by Barrozo et al. using the ωB97X-D functional in Gaussian 09.^23,39^ In the present work, α-β-γ refers to a binding mode in which magnesium associates with the α-, β-, and γ-phosphoryl groups of methyl triphosphate. β-γ refers to a binding mode in which magnesium associates with the β- and γ-phosphoryl groups of methyl triphosphate. These molecular systems all have an explicit solvent shell of 17 water molecules in addition to the one lytic water molecule that performs the nucleophilic attack.^23^ The literature reactants and products underwent further unconstrained optimization in ORCA using the tightopt and then verytightopt convergence thresholds. The final optimized reactant and product states underwent vibrational frequency calculations to ensure that no imaginary vibrational modes existed and to provide the thermochemical properties of each system.

### Transition State Searches

Transition state searches were completed using the NEB-CI method to obtain a transition state guess.^34,53^ The NEB-CI calculation converged 8 images along the minimum energy path between our fully optimized reactant and product states using L-BFGS optimization and the default NEB-CI convergence thresholds.^53,67^ The converged climbing image from the NEB-CI calculation served as the initial structure for the first eigenvector-following transition state optimization. The resulting structure was used as the input for the second eigenvector-following calculation with tighter convergence thresholds, corresponding to the verytightopt keyword. The eigenvector-following calculations (OptTS keyword) used an initial analytical Hessian, tight SCF convergence thresholds (TightSCF keyword), and slow convergence (SlowConv keyword). The final optimized transition state underwent a vibrational frequency calculation to verify the presence of only a single imaginary vibrational mode and to obtain thermochemical properties.

### Metaphosphate and Phosphorane Intermediates

To further evaluate the nature of the phosphoryl transfer mechanism, we attempted to construct metaphosphate and phosphorane states. The previously optimized metastable product state with magnesium in the α-β-γ mode was perturbed through constrained geometry optimizations. Each constrained optimization was carried out using tightopt and then verytightopt convergence thresholds. The coordinates from verytightopt optimizations (whether constrained or unconstrained) underwent vibrational frequency calculations. To perturb toward a metaphosphate, we optimized while imposing a bond angle constraint of 120.0° between nonbridging oxygens on the γ-phosphoryl group, and a dihedral angle constraint of 0.0° between these three nonbridging γ-substituent oxygens and the γ-phosphorus. To perturb toward a phosphorane, we optimized the metastable product state while imposing distance constraints of 1.68 Å between the γ-phosphorus and the leaving oxygen and the γ-phosphorus and attacking oxygen. To test the stability of phosphoranes, they were reoptimized through unconstrained geometry optimizations at the tightopt and then verytightopt convergence thresholds.

### Mg·MeTP^2–^ Hydrolysis in Alternative Solvents

To ascertain the forces behind concerted vs dissociative bond orders in the transition state, we generated artificial systems designed to alter the electrostatic and steric properties of the solvent environment. Specifically, Mg·MeTP^2–^ hydrolysis was studied in two lower dielectric chemical environments: SMD implicit acetone and SMD implicit water with the dielectric set equal to that of acetone (20.4930). We applied the following workflow with each of these respective chemical environments. Just as when characterizing aqueous hydrolysis reactions, we started with the coordinates from Barrozo et al. corresponding to the reactants and products of solvent-assisted hydrolysis of Mg·MeTP^2–^ with magnesium bound in “mode 3” (the α-β-γ mode).^23,39^ All explicit water molecules were removed except the lytic water and the assisting water (the water receiving a hydrogen bond from the lytic water). The reactants underwent unconstrained geometry optimization in ORCA at tightopt, then verytightopt convergence thresholds. The products first underwent optimization at the looseopt convergence threshold, with the distance between the γ-phosphorus and the leaving oxygen constrained at 3.60 Å to avoid relaxation into the reactant state. Subsequently, the products underwent unconstrained optimization at the normalopt, tightopt, and verytightopt convergence thresholds. These optimized reactants and products served as the input start and end states in a NEB-CI run.^53^ The NEB-CI run for the system in water with a custom acetone dielectric could not converge fully after 1500 iterations, because the RMS­(FCI) and MAX­(|FCI|) were too high at 0.001008 Eh·Bohr^–1^ and 0.005946 Eh·Bohr^–1^, respectively (see Supporting Information for convergence thresholds). However, the climbing image had a single imaginary vibrational mode and underwent eigenvector-following optimizations to an energetic saddle point, to complete the transition state search. Other than the above issue with NEB-CI convergence, these systems were treated with the same protocols as described in “Transition State Searches” above. To evaluate the effect of basicity on the transition state, we reoptimized the transition state previously optimized in implicit acetone, increasing solb to 0.90 (the default for acetone is 0.49). During this reoptimization, the other SMD implicit solvation parameters were kept at the defaults for acetone.

## Results and Discussion

In our attempt to approximate nucleoside triphosphate hydrolysis, we use methyl triphosphate to balance computational efficiency with accuracy.^21,23,26^ Modeling a nucleoside moiety is likely unnecessary as both NMR and *in vitro* vibrational spectra suggest it interacts negligibly with the magnesium counterion near physiological pH.^68,69^ We attempt to comprehensively describe the mechanism using interatomic distances and bond orders. We contextualize our characterization of transition states with respective reactants and products to describe how much bond formation and bond breaking happens before and after the formation of the transition state.

### Binding Modes of Magnesium to Methyl Triphosphate

Figure 1 shows the nomenclature for the three phosphoryl groups in methyl triphosphate. α-β-γ refers to a binding mode in which magnesium associates with the α-, β-, and γ-phosphoryl groups of methyl triphosphate. β-γ refers to a binding mode in which magnesium associates with the β- and γ-phosphoryl groups of methyl triphosphate. Most computational studies seeking to characterize the hydrolysis pathways of Mg·MeTP^2–^ or Mg·ATP^2–^ account for a single Mg^2+^ binding mode, usually involving the β-γ mode,^21,22,24–26^ but in at least one case the α- and γ-phosphoryl groups.^27^ These approaches were followed despite NMR and *in vitro* vibration analysis evidence for magnesium binding to ATP primarily in the β-γ and α-β-γ modes near physiological pH.^70,71^ In contrast, Barrozo et al. characterized the transition states of Mg·MeTP^2–^ hydrolysis while accounting for three different Mg^2+^ binding modes to the phosphoryl groups of MeTP^4–^: β-γ (“mode 1”); α-β (“mode 2”); and α-β-γ (“mode 3”).^23^ Like Barrozo et al., we consider the experimentally predominant β-γ and α-β-γ modes in our investigation of aqueous Mg·MeTP^2–^ hydrolysis.^70,71^ Not only do these modes predominate in solution, but also in proteins binding ATP. In 2021, Buelens et al. reported a survey of 2123 Mg·ATP configurations in which 49.7% had magnesium bound in a β-γ mode and 27.5% in an α-β-γ binding mode.^72^

Thermochemistry calculations on the reactant states (Figure 2, top row) predicted that Mg^2+^ association with the phosphoryl groups of MeTP^4–^ is more favorable in an α-β-γ configuration than a β-γ configuration. At a temperature of 298.15 K, this favorability is predicted by a Gibbs free energy difference of −3.6 kcal·mol^–1^. This Gibbs free energy difference is partly driven by an enthalpy difference of −1.5 kcal·mol^–1^ that may result from a more complete charge pairing between MeTP^4–^ and Mg^2+^ when all three phosphoryl groups bind the magnesium counterion. The binding of magnesium to all three phosphoryl groups also has an even greater entropic favorability with a *T*Δ*S* of 2.1 kcal·mol^–1^. This entropic favorability is 99.5% attributable to a difference in vibrational entropy (with 0.5% rotational contribution), suggesting a greater multiplicity of vibrational modes when binding Mg^2+^ in the α-β-γ mode instead of the β-γ mode. A possible explanation for this entropic favorability is that in the α-β-γ magnesium binding mode, the magnesium and α-phosphoryl group have fewer sites for electrostatic interaction with the aqueous solvent, and therefore place less constraint on the vibrational motion of the solvent. Electrostatic interaction with Mg·MeTP^2–^ would constrain the motion of water molecules more than interaction with other water molecules because of the inflexibility of Mg·MeTP^2–^ complex compared to a network of liquid phase water molecules. This hypothesis conceptually agrees with experimental binding calorimetry data suggesting an entropically favorable release of water from the hydration shells around pyrophosphate and ATP.^73,74^ The predicted thermodynamic advantage of binding magnesium to all three phosphoryl groups is worth noting because this binding mode has often been ignored by recent QM studies of Mg·MeTP^2–^ or Mg·ATP^2–^ hydrolysis.^21,22,24–26^

**Figure 2:**
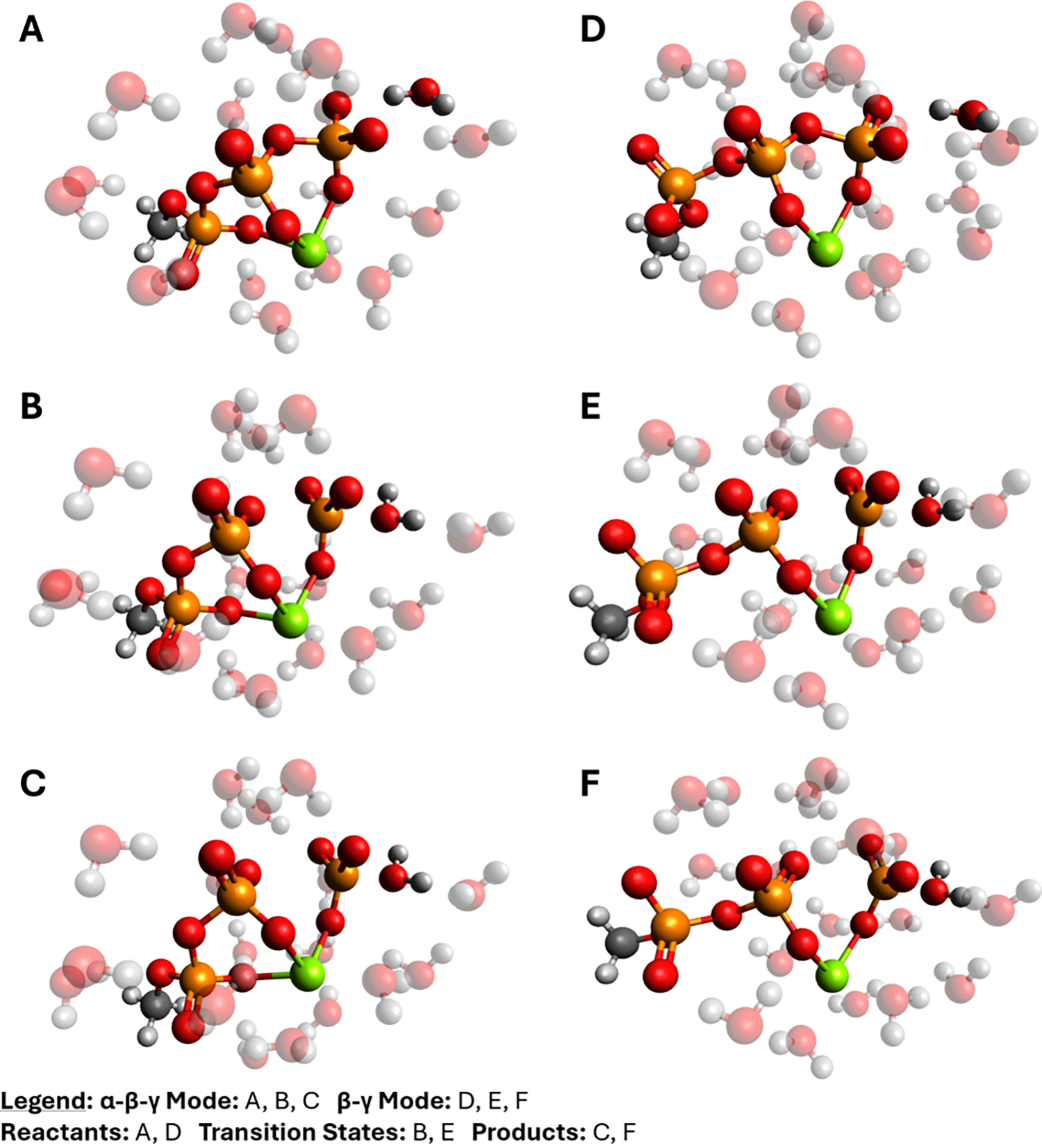
Optimized states for phosphoryl transfer from Mg·MeTP^2–^ to water, in the α-β-γ (A, B, C) and β-γ (D, E, F) magnesium binding modes: the reactant states (top; A, D), the transition states (middle; B, E), and the product states (bottom; C, F). The magnesium counterion is colored green; phosphorus, orange; oxygen, red; carbon, gray; and hydrogen, white. All solvent molecules are faded except the lytic water.

### Aqueous Transition States

The calculation of vibrational modes for a given chemical system plays a critical role in our mechanistic analysis. The absence of imaginary vibrational modes indicates close proximity to an energetic minimum. An imaginary vibrational mode arises when the internuclear geometry of a chemical system changes while passing over an energy barrier, sampling motion along the reaction path toward both reactants and products.^75^ In the case of a transition state, the motion samples the reactants and products equally well. For example, in the phosphoryl transfer, the modes of a concerted reaction would show equal sampling toward methyl triphosphate on one side of the activation barrier and methyl diphosphate + orthophosphate on the other side of the barrier. Alternatively, a dissociative reaction would show equal sampling toward methyl triphosphate on one side of the activation barrier and methyl diphosphate + a dissociated trigonal planar PO_3_ (metaphosphate) on the other side of the barrier.

The optimized transition state for the α-β-γ mode had a single imaginary vibrational frequency of −214.66 cm^–1^. The transition state for the β-γ mode had a single imaginary vibrational frequency of −168.80 cm^–1^. When animated, each imaginary vibrational mode shows a water molecule performing a nucleophilic attack on the phosphorus center of the γ-phosphoryl group of Mg·MeTP^2–^. In both cases, the nucleophilic attack abstracts this phosphorus center away from the bridging oxygen. Figure 2 shows the structures of these transition states along with respective reactant and product states.

### Thermodynamic Barriers to Hydrolysis

Table 1 contains our *in silico* thermodynamic quantities related to Mg·MeTP^2–^ hydrolysis and, for comparison, Table 2 experimental values from kinetic studies of Mg·ATP and Mg·GTP hydrolysis. The *in silico* Δ*G* ^‡^ is lower in the α-β-γ mode (27.5 kcal·mol^–1^) than in the β-γ mode (30.1 kcal·mol^–1^). The Δ*G* ^‡^ in the α-β-γ mode is also more consistent with the cited experimental values for the Δ*G* ^‡^ of nonenzymatic Mg·ATP and Mg·GTP hydrolysis: 27.5–27.6 and 27.9 kcal·mol^–1^, respectively.^41–43,65^ These comparisons suggest that Mg·ATP and Mg·GTP hydrolysis preferentially proceed with magnesium bound to all three phosphoryl groups. This result indicates QM studies of Mg·MeTP^2–^ or Mg·NTP^2–^ hydrolysis must include this α-β-γ binding mode, despite it typically being ignored.^21,22,24–26^ These studies have calculated Δ*G* ^‡^ values in the range of 29–35 kcal·mol^–1^ when magnesium is bound in the β-γ mode, which is outside the range of the *in vitro* measurements.^21,22,24–26^ Barrozo and colleagues’ DFT study of Mg·MeTP^2–^ hydrolysis predicted a similar pattern of Gibbs free energy barriers in relation to magnesium binding mode: 29.2 kcal·mol^–1^ when Mg^2+^ is bound in the α-β-γ mode, and 30.6 kcal·mol^–1^ when Mg^2+^ is bound in the β-γ mode. The improved accuracy of our predicted Gibbs free energy barrier can be reasonably attributed to two improvements in the level of theory. The first is the DFT-D4 dispersion correction with TD-DFT-generated polarizabilities, and dependence on both atomic charge and system geometry in contrast to the adaptation of the D2 scheme in ωB97X-D.^23,35–37,39^ The other improvement is performing geometry optimizations and vibrational frequency calculations using the 6-311++G­(d,p) basis set, allowing for more accurate handling of hydrogen bonding than 6-31+G­(d).^23,55^

**Table 1:** *In Silico* Thermodynamics from Reactants to Transition States^a^

Mg^2+^ binding mode	solvent	temp (K)	Δ*G* ^‡^ (kcal·mol^–1^)	Δ*H* ^‡^ (kcal·mol^–1^)	*T*Δ*S* ^‡^ (kcal·mol^–1^)
α-β-γ	water^b^	298.15	27.5	27.0	–0.5
		333.15	27.5		
	custom implicit water^c^	298.15	23.7	23.6	–0.1
	implicit acetone^c^	298.15	26.3	26.9	0.7
β-γ	water^b^	298.15	30.1	29.4	–0.7
		333.15	30.2		

a Activation Gibbs free energies, enthalpies, and entropies multiplied by temperature pertaining to the *in silico* nonenzymatic phosphoryl transfer from Mg·MeTP^2–^ to water.

b ε = 78.3550.

c ε = 20.4930.

**Table 2:** *In Vitro* Thermodynamics from Reactants to Transition States^a^

temp (K)	pH	nucleotide	Δ*G* ^‡^ (kcal·mol^–1^)	Δ*H* ^‡^ (kcal·mol^–1^)	*T*Δ*S* ^‡^ (kcal·mol^–1^)
298	6.0–8.0	Mg·ATP	27.5 ^42^	25.6 ^42^	–1.9 ^42^
	[unknown]	Mg·GTP	27.9 ^41^	27.1 ^41^	–0.8 ^41^
333	7	Mg·ATP	27.6 ^43^		

a Activation Gibbs free energies, enthalpies, and entropies multiplied by temperature pertaining to experimental nonenzymatic phosphoryl transfer to water from Mg·ATP or Mg·GTP. Gibbs free energy values for 298.15 K were calculated using an Arrhenius plot extrapolation of the rate constant. When Gibbs free energies of activation were calculated from rate constants using transition state theory, the transmission coefficient was taken as unity.

In our systems, the Gibbs free energy barriers are mostly enthalpic, with a Δ*H* ^‡^ of 27.0 kcal·mol^–1^ for the α-β-γ mode and 29.4 kcal·mol^–1^ for the β-γ mode (Table 1). As with the Gibbs free energy barriers, the enthalpy barrier is lower and more experimentally accurate in the α-β-γ mode, with the experimental Δ*H* ^‡^ value for Mg·GTP hydrolysis 27.1 kcal·mol^–1^ and Mg·ATP hydrolysis 25.6 kcal·mol^–1^.^41,42^ The difference between our *in silico* enthalpy barriers may result from more energetically favorable electron transfer away from the P_γ_–O_l_ bond when all three phosphoryl groups are charge-paired with Mg^2+^ instead of only the β- and γ-phosphoryl groups. Our enthalpy barrier of 27.0 kcal·mol^–1^ in the α-β-γ mode is closer to that reported by Kötting and Gerwert for Mg·GTP hydrolysis (27.1 kcal·mol^–1^),^41^ than by Stockbridge and Wolfenden for Mg·ATP hydrolysis (25.6 kcal·mol^–1^).^42^ and likewise for the entropies multiplied by temperature (Table 1).^41,42^ The lack of clarity from Kötting and Gerwert regarding the pH and magnesium concentration at which Mg·GTP hydrolysis was studied suggests the agreement might improve with adjustments to those parameters.

### Metastable Product States for Aqueous Hydrolysis

The metastable intermediates define the product states of the aqueous-solvated processes characterized in this study. We do not attempt to characterize hydrolysis beyond the formation of this metastable intermediate partly because Glaves et al., in their *ab initio* metadynamics characterization of Mg·MeTP^2–^ hydrolysis,^21^ found the barrier between this state and the reactant state determines the reaction rate. Later states involve maturation of the phosphate conformation, proton transfer, and hydration, all of which do not impinge upon the phosphoryl transfer mechanism. The metastable intermediates we found have Gibbs free energies which are only 2.7 kcal·mol^–1^ (α-β-γ mode) and 2.4 kcal·mol^–1^ (β-γ mode) less than those of their respective transition states, consistent with the results of Glaves et al. (free energy change of ∼ −2 kcal·mol^–1^ between the first transition state and the following intermediate state). In both magnesium binding modes, the geometry of the metastable intermediate shows significant association with the attacking oxygen (1.83–1.87 Å) and dissociation from the leaving oxygen (3.02–2.93 Å). Likewise, bond orders show a significant association of the phosphorus with the attacking oxygen (P_γ_–O_nuc_ bond order 0.60–0.61) and dissociation from the leaving oxygen (P_γ_–O_l_ bond order 0.16–0.09). Therefore, these metastable states do not appear as metaphosphates or phosphoranes, but as the incipient monophosphate product.

### Mechanistic Characterization of Aqueous Hydrolysis

Having established the consistency of the model with experimental data, we turned our attention to understanding the reaction mechanism. The differences in aqueous reactant state and transition state Gibbs free energies both favor the α-β-γ tridentate binding mode over the β-γ bidentate mode, so we down-prioritize discussion of the β-γ bidentate mode from this point. Reaction mechanisms can occur as associative (full bonds formed between the electrophile and both the leaving group and nucleophile), dissociative (no bonds between the electrophile and both leaving group and nucleophile), or concerted (half-bonds between electrophile and both leaving group and nucleophile.

When magnesium binds to aqueous MeTP^4–^ in an α-β-γ tridentate configuration, the reaction appears concerted as indicated by interatomic distances in Table 3, but with bond orders implying some late (dissociative) character (Table 4). Most of the spatial association between the attacking nucleophile and the phosphorus center (internuclear distance going from 3.60 to 2.13 Å) happens before the formation of the transition state, rather than afterward (going from 2.13 to 1.83 Å). However, the bond orders show less electronic association between the attacking nucleophile and the phosphorus center before the formation of the transition state (0.12 to 0.32) compared to afterward (0.32 to 0.60). Therefore, the attacking oxygen has completed most of its spatial approach when it has donated enough electrons for the system to reach an energetic saddle point. However, even at this saddle point, electron donation from the attacking nucleophile is estimated as only modest (bond order 0.32).

**Table 3:** Interatomic Distances for Mechanistic Characterization^a^

	interatomic distance (Å)					
	water^b^		custom implicit water^c^		implicit acetone^c^	
state	P_γ_–O_l_	P_γ_–O_nuc_	P_γ_–O_l_	P_γ_–O_nuc_	P_γ_–O_l_	P_γ_–O_nuc_
reactants	1.68	3.60	1.69	3.72	1.70	3.68
transition state	2.36	2.13	2.39	2.28	2.59	2.42
products	3.02	1.83	4.21	1.68	4.94	1.94

a Interatomic distances (Å) relevant to the *in silico* phosphoryl transfer to water from Mg·MeTP^2–^ in the α-β-γ Mg^2+^ binding mode.

b ε = 78.3550.

c ε = 20.4930.

**Table 4:** Mayer Bond Orders for Mechanistic Characterization^a^

	Mayer bond order					
	water^b^		custom implicit water^c^		implicit acetone^c^	
state	P_γ_–O_l_	P_γ_–O_nuc_	P_γ_–O_l_	P_γ_–O_nuc_	P_γ_–O_l_	P_γ_–O_nuc_
reactants	0.71	0.12	0.70	0.01	0.88	0.04
transition state	0.27	0.32	0.15	0.30	0.08	0.25
products	0.16	0.60	–0.02	0.94	0.01	0.56

a Mayer bond orders relevant to the *in silico* phosphoryl transfer to water from Mg·MeTP^2–^ in the α-β-γ Mg^2+^ binding mode.

b ε = 78.3550.

c ε = 20.4930.

The spatial dissociation from the leaving oxygen occurs steadily; the interatomic distance between P_γ_ and O_l_ increases very evenly by 0.68 Å before and 0.67 Å after the transition state. Simultaneously, P_γ_–O_l_ bond order decreases mostly before the transition state (0.71 to 0.27) rather than afterward (0.27 to 0.16). This change in bond order suggests the local electronic integrity of the P_γ_–O_l_ bond is poorly maintained even with only a modest donation of electrons from the attacking oxygen to the γ-phosphorus, and partial spatial dissociation from the leaving phosphoryl group.

The sum of P_γ_–O_l_ and P_γ_–O_nuc_ bond orders is only 0.59 at the transition state but 0.83 and 0.75 for the reactants and products, respectively. Some overlap between P_γ_–O_l_ bond cleavage and P_γ_–O_nuc_ bond formation exists, but the former tends to precede the latter, suggesting some late character. However, the animated imaginary vibrational mode across the energetic saddle point shows a smooth continuous transition from methyl triphosphate, through a transition state with methyl diphosphate and associated trigonal planar PO_3_ group, to methyl diphosphate and a nascent orthophosphate. Therefore, the mechanisms of nonenzymatic methyl triphosphate, and by implication Mg·NTP^2–^, hydrolysis is expected to be concerted, despite bond orders suggesting dissociative/late pathway character.^1^ The late bond orders for the Mg·MeTP^2–^ hydrolysis pathway and transition state might be explained at least partly by electrostatic repulsion. In all three systems in Tables 3 and 4, the attacking oxygen has a weaker negative charge (∼−0.15 to −0.20) compared to the leaving oxygen (∼−0.65 to −0.80). The trigonal planar PO_3_ group formed from the γ-phosphoryl group also has a net negative charge of ∼−0.61 to −0.71. Therefore, this PO_3_ group experiences electrostatic repulsion with both the attacking and leaving oxygen, but more so with the leaving oxygen. The differential repulsion correlates with longer P_γ_–O_l_ bond distances and weaker bond orders relative to those for P_γ_–O_nuc_.

### Phosphorane and Metaphosphate Intermediates

We explicitly tested for the possibility of phosphorane and metastable intermediates by modeling them *in silico*. We successfully produced a phosphorane geometry through constrained optimizations that forced O_l_ and O_nuc_ to remain at a proper single bond distance to the P_γ_. However, the resulting system did not reside at an energetic minimum, having two imaginary vibrational modes at −77.14 cm^–1^ and −21.37 cm^–1^. Once the geometric constraints were removed, the phosphorane relaxed into methyl triphosphate. Moreover, our thermochemistry calculations show that the phosphorane geometry has a Gibbs free energy of 61.0 kcal·mol^–1^ greater than the reactant state, and 33.5 kcal·mol^–1^ greater than our transition state. Therefore, we conclude that a phosphorane species is excluded as a potential intermediate in Mg·NTP^2–^ hydrolysis mechanisms. Our attempt to optimize a metaphosphate state was unsuccessful because even when a planar geometry was enforced for the γ-phosphoryl group, the γ-phosphorus was still too closely associated with the leaving oxygen (2.20 Å) and the attacking oxygen (2.30 Å). The result was a structure similar to the transition state (see interatomic distances in Table 3). Vibrational analysis of this structure indicated that it was not at an energetic minimum, having an imaginary vibrational mode at −243.27 cm^–1^. Therefore, we also judge that a long-lived metaphosphate species is an unlikely intermediate in Mg·NTP^2–^ hydrolysis. The sum of this analysis implies that the hydrolysis of Mg·NTP^2–^ species does not proceed through a stepwise associative pathway or stepwise dissociative pathway with experimentally observable metaphosphate.^1^ Our attempts to model phosphorane and metaphosphate geometries demonstrated that these states are unstable relative to the concerted pathway.

### Mg·MeTP^2–^ Hydrolysis in Alternative Solvents

Most of the evidence that we have presented supports a concerted pathway of aqueous Mg·MeTP^2–^ hydrolysis, except for the bond orders which suggest some dissociative or late character. Assuming the Mg·MeTP^2–^ hydrolysis follows a concerted pathway, we can probe the physical properties driving the overly low predicted transition state bond orders. As observed earlier, electrostatic repulsion correlates with bond order. Therefore, we create artificial systems that should alter the behavior of the transition state in accordance with altered electrostatic interactions. To accomplish this, we studied Mg·MeTP^2–^ hydrolysis in two alternative solvent conditions. To investigate the effects of increased electrostatic interactions in a medium otherwise equivalent to an aqueous one, we searched for the transition state using SMD implicit water with a custom dielectric set equal to that of acetone. In this model, most of the explicit solvent, except the attacking and assisting water molecules, was removed to better control system polarizability. If the bond orders in the transition state are driven by electrostatic repulsion, then lower bond orders and larger bond distances between the planar PO_3_ group and the attacking and leaving oxygen atoms should occur in a lower dielectric environment. The other solvent condition involved SMD implicit acetone, which should enhance the concerted nature of the reaction. similar to S_N_2 reactions involving organic scaffolds, but which may enhance the electrostatic repulsion because of the lack of hydrogen bonding to the phosphate oxygens.

Compared to ordinary SMD water with an explicit water shell, the water with a custom dielectric resulted in a pathway with greater disparity in bond orders between reactants and products on one hand and the transition state on the other, implying greater late character (Table 4). The P_γ_–O_l_ bond order in the reactant state is nearly the same in both solvents (0.71 and 0.70). In the lower dielectric solvent, it decreases to only 0.15 at the transition state, whereas in ordinary water it only decreases to 0.27. The P_γ_–O_nuc_ bond orders are nearly equal in either solvent (0.30 and 0.32, in low and high dielectrics, respectively) at the transition state. Compared to the other two solvent conditions, SMD acetone produces a pathway with more late character in that the P_γ_–O_l_ and P_γ_–O_nuc_ bond distances are greater (Table 3) and the P_γ_–O_l_ and P_γ_–O_nuc_ bond orders are weaker (Table 4). Because the implicit acetone parametrization uses a nonzero basicity constant for oxygen while none for water, we reoptimized the acetone-solvated transition state in custom SMD acetone with greater basicity by increasing solb from 0.49 to 0.90. However, the resulting transition state in the custom acetone had nearly the same P_γ_–O_l_ and P_γ_–O_nuc_ bond lengths as in ordinary acetone (2.60 Å and 2.44 Å respectively), and nearly the same P_γ_–O_l_ and P_γ_–O_nuc_ bond orders (0.08 and 0.25 respectively) as in ordinary acetone (Tables 3 and 4). Therefore, the specific polar electrostatic properties of the solvent appear to have little effect on the transition state beyond the global effects from the dielectric. Given that the Mayer bond orders lack incorporation of the effects of diffuse orbitals, the actual bond orders are likely larger than those reported in Table 4 and Table S2. By varying dielectric and solvent identity, we have evidence that the weaker predicted bond orders at the transition state likely arise from increased electrostatic repulsion and possibly increased average steric bulk from the solvent. Nevertheless, the overlaps from diffuse orbitals help maintain a concerted reaction dynamic through the transition state.

Despite the decreased combined P_γ_–O_l_ and P_γ_–O_nuc_ bond order at the transition state, other aspects of the Mg·MeTP^2–^ hydrolysis transition state suggest a concerted mechanism. The trends in bond orders and bond distances show continuous transitions from a reactant state to a transition state to a product state that has either a nascent orthophosphate or a completely formed orthophosphate, but not a phosphorane or metaphosphate. These bond orders and bond distances also show overlaps between bond breaking and bond formation at the transition states of these processes. Lastly, animation of the imaginary vibrational mode at each transition state shows a smooth, continuous transition from a reactant-like methyl triphosphate structure through the transition state, to a product state with a nascent cleaved phosphate. Therefore, we conclude that aqueous Mg·MeTP^2–^ hydrolysis is concerted but has late (dissociative-looking) bond orders because of poorly incorporated contributions from diffuse orbitals and possibly steric effects from the solvent.

Because both bond length elongation/contraction between thermodynamic end points and the transition state or bond orders have been considered measures of where on the two-dimensional reaction space the transition state lies, we calculated percent progression of bond lengths and orders at the transition state (Table 5).

**Table 5:** Percent Progression of Relevant Bonds for Mechanistic Characterization^a^

	water^b^		custom implicit water^c^		implicit acetone^c^	
transition state property	P_γ_–O_l_	P_γ_–O_nuc_	P_γ_–O_l_	P_γ_–O_nuc_	P_γ_–O_l_	P_γ_–O_nuc_
interatomic distance, percent progression	50.75	83.05	27.78	70.59	27.47	72.41
Mayer bond order, percent progression	80.00	41.67	76.39	31.18	91.95	40.38
total	130.75	124.72	104.17	101.77	119.42	112.79
difference between P_γ_–O_l_ and P_γ_–O_nuc_	6.03		2.40		6.63	

a Percent progression of interatomic distances and Mayer bond orders, their combined totals for each bond, and differences between percent progression for the P_γ_–O_l_ and P_γ_–O_nuc_ bonds for each transition state. These values pertain to the transition states of *in silico* phosphoryl transfer to water from Mg·MeTP^2–^ in the α-β-γ Mg^2+^ binding mode.

b ε = 78.3550.

c ε = 20.4930.

While bond-breaking progression is quite pronounced, bond formation appears slightly under-formed relative to a naïve expectation of 50%. On the other hand, the interatomic distances show significant retardation on the leaving side and a highly significant approach past the halfway mark for the nucleophilic water at the transition state. While neither measure suggests the balance expected of a coordinated transition, and superficially implies looseness on one side of the reaction or the other, the sums of the fractional progressions are nearly identical. The differences in value between sums are small, suggesting a potential measure for concertedness. For symmetric reactions, where the leaving group and nucleophile are the same, this criterion automatically is satisfied. Unfortunately, for asymmetric systems, both bond orders and all necessary bond lengths are incompletely available, so confirming the generality of this criterium is more challenging. We are unaware of any work on asymmetric S_N_2 reactions that report both bond orders and interatomic distances for the reactant, transition state, and product, preventing further validation of this metric.

## Conclusions

We explored the hydrolysis of methyl triphosphate into methyl pyrophosphate and orthophosphate seeking to clarify magnesium binding preference over the course of the reaction and to better understand the nature of the S_N_2 transition state. Through the application of the ωB97X-D4 functional and SMD implicit solvation model,^56^ we have gained precise control of the electrostatic and steric environment for reactant, product, and transition states. For the reactant state, we observed that magnesium prefers the α-β-γ mode over the β-γ mode. This preference extends to the transition state, where the predicted barrier is also lower. The predicted Gibbs free energy barrier for the α-β-γ mode adheres quite closely to experimental results.^41–43^ Therefore, we advise that future computational investigations of the hydrolysis of Mg·MeTP^2–^ or a given Mg·NTP^2–^ account for the binding mode of magnesium to all three phosphoryl groups.

Bond orders, bond distances, and the animation of imaginary vibrational modes support a concerted mechanism for the hydrolysis of Mg·MeTP^2–^, a model system for biochemically relevant Mg·NTP^2–^ species. Bond orders suggest some late transition state character, but no stepwise associative or dissociative mechanism. The concerted mechanism appears in implicit water with an explicit water shell; implicit water with an acetone dielectric; and implicit acetone solvent conditions. A late, or “loose” concerted reaction pathway (overly reduced bond orders in the transition state) is consistent with the *in silico* DFT characterization of solvent-assisted Mg·MeTP^2–^ hydrolysis reactions by Barrozo et al.^23^ In contrast, a dissociative pathway has been suggested by the metadynamics studies of Mg·MeTP^2–^ hydrolysis by Glaves et al. and Mg·ATP^2–^ hydrolysis by Saxena et al.^21,25^ A concerted transition state with dissociative character is consistent with Bro̷nsted correlational analysis of Admiraal and Herschlag.^33^ However, we suggest that the seemingly dissociative character of this process is a result of overweighting electrostatic repulsion between the PO_3_ group and the attacking and leaving oxygen atoms. The repulsion decreases traditional σ bond strength but leads to a more diffuse electronic structure. This diffuse character results in an underestimation of bond orders for the transition state. Lowering the dielectric created more repulsion, longer bond distances, and smaller bond orders. Yet one bond breaks as a new one forms and the transition state imaginary mode shows a transfer of the PO_3_ group from the triphosphate to the water, consistent with only a concerted mechanism. We cannot overstate the importance of this observation. In a “loose” transition, especially in cases like the triphosphate hydrolysis where one bond models as essentially broken in the transition state, one might expect that a potentially small fraction of the time, an S_N_1-like transition should occur, with the formation of metaphosphate as a short-lived intermediate. We find that metaphosphate does not arise, but we cannot exclude the possibility that certain specific protein environments could stabilize the metaphosphate.^76,77^ Rather, the interpretation of the bond orders and bond stretching/contraction should reflect the known S_N_2 outcome. Considering the weak transition state bond orders, but the lack of evidence for a metaphosphate, the term “dissociative” should be reserved for S_N_1 reactions. Instead, the term “late” is more appropriate as it leans on the Hammond-Leffler postulate of mimicry of the product more than the reactant. This term makes no assertion about the mechanism and hence still allows for a fully concerted reaction.

Our results suggest that S_N_2 mechanisms can sometimes apply to late transition state processes with dissociative-looking Mayer bond orders. Mayer bond orders are insufficient to determine the reaction mechanism under charged conditions because they improperly account for diffuse functions in the basis set. However, the extent to which electron delocalization and diffusivity affect a given system is unclear and cannot be addressed in a general sense here. Nevertheless, the considerable decrease in the KIE ratio in going from mono- to di- to triphosphoesters suggests that a decreased capacity in sharing electrons diffusively forces greater concentration of the electron density along the reaction axis, resulting in the appearance of more phosphorane character in the transition state.^1^ A neutral system, where repulsion would be minimal, may be more suited for analysis with Mayer bond orders. Yet when we consider both the fractional change in bond lengths as well as in bond orders in combination, the outcomes suggest nearly equal behaviors between the nucleophile and the leaving group for concerted S_N_2 reactions.

What criteria appear necessary for a prediction of an S_N_2 concerted reaction based on models of reactants, products, and the intervening transition state? The inclusion of some sort of solvent effects appears as a necessity based on earlier works.^3,4,23,78^ The motions from the transition state imaginary vibrational mode must be consistent with the nascent formation of both reactant and product geometries. Further, the sum of the fraction progression from reactant to product bond lengths and orders at the transition state should be nearly equal for the bonds to both the nucleophile and the leaving group. Changing the identity of the implicit solvent from water to acetone, we also conclude that the steric bulk of the solvent might further weaken the P_γ_–O_l_ and P_γ_–O_nuc_ bonds at the transition state. However, varying basicity during transition state optimization had little effect on relevant bond distances or bond orders, suggesting negligible importance of solvent electrostatic properties beyond its overall dielectric. Since the bond orders appear to vary with the intensity of the electrostatic repulsion (as a function of the dielectric), these diffuse and polarizable components in the theoretical description appear quite significant. More importantly, the diffuse nature of the transition state bonding implies stronger correlated motion between the electrons in the reacting oxygens and the PO_3_ group. Hence, representations likely should include diffuse and polarizable components as well. We anticipate that QM studies implementing these criteria will provide more reliable mechanistic characterizations.

More generally, substitution reactions on highly oxidized centers likely also proceed through a similar concerted mechanism. The attacking and leaving groups will both be nucleophilic, while the oxidized center will be strongly electrophilic. The groups attached to the electrophile will also be nucleophilic, leading to electrostatic repulsion in the transition state between all groups. Nevertheless, as long as diffuse orbitals, presumably on the central electrophilic atom, can be occupied at relatively low energetic cost, the total bond strength can be maintained, leading to a concerted reaction mechanism.

Given that the predicted mechanism for phosphoryl cleavage appears concerted and independent of solvent electrostatic and steric properties, and therefore a general feature of triphosphate cleavage, we assert that the mechanism would still hold in protein environments. Since protein pockets have predicted effective dielectrics near that of acetone (∼20–30),^79^ most of the calculations we performed should transfer immediately, provided similar geometries are observed. Our study does not support the greater favorability of the β-γ mode in an aqueous environment. However, certain proteins could have also evolved to induce a β-γ binding mode so that both the magnesium ion and the α-phosphoryl group participate in more electrostatic pairing with protein polar groups than would be possible in the more compact, less exposed α-β-γ magnesium configuration. These alternate contacts would aid the binding of the ATP molecule long enough for catalysis to take place. Our investigation of Mg·MeTP^2–^ hydrolysis in implicit water with a custom acetone dielectric predicted a lower activation barrier compared to ordinary water. In this custom solvent, the Δ*H* ^‡^ was only 23.6 kcal·mol^–1^ and the Δ*G* ^‡^ 23.7 kcal·mol^–1^ (Table 1). In contrast, in implicit acetone, these values change to a Δ*H* ^‡^ of 26.9 kcal·mol^–1^ and a Δ*G* ^‡^ of 26.3 kcal·mol^–1^(Table 1). Thus, a lower dielectric correlates with a decreased enthalpy and free energy barrier. However, steric effects from the bulkier acetone solvent may have increased this enthalpy barrier. Therefore, we expect that a lower dielectric and a lack of steric interference contribute to the catalytic properties of enzymatic phosphoryl transfer active sites. Positively charged side chains such as lysine and arginine may function to hold the triphosphate moiety of Mg·NTPs in place to ensure a favorable orientation relative to the substrate receiving the phosphoryl transfer. However, the protein Ras lowers the activation enthalpy of GTP hydrolysis from 27.1 to 19.8 kcal·mol^–1^, a significantly greater catalytic effect than we achieved by lowering the dielectric of implicit water.^41^ Therefore, the present work cannot reasonably exclude the possibility that specific enzyme–substrate interactions lower the enthalpy barrier of Mg·NTP^2–^ hydrolysis reactions.

The catalytic effect from a lowered dielectric could be the result of increased electrostatic repulsion between the negatively charged β- and γ-phosphoryl groups in the reactant state in a lower dielectric. In this case, the lower dielectric of protein cavities helps destabilize the reactant state. The planar PO_3_ group in the transition state would also experience greater electrostatic repulsion with the attacking and leaving oxygen atoms, leading to a transition state with larger P_γ_–O_l_ and P_γ_–O_nuc_ distances, just as we observed when we lowered the dielectric of implicit water. However, it is alternatively possible that such electrostatic repulsion is alleviated by positively charged biochemical moieties such as lysine and arginine. In this case, the transition state would be stabilized. A third factor to consider is how enzymes assist the flow of electrons through the substrates involved in phosphoryl transfer. Charge pairing between triphosphate moieties of NTPs and positively charged groups within the protein may help draw electrons away from the P_γ_–O_l_ bond, assisting its cleavage.^41^ This concept is already tentatively supported by our lower activation enthalpy when all three phosphoryl groups of Mg·MeTP^2–^ are paired with magnesium, instead of only the β- and γ-phosphoryl groups. Negatively charged moieties on the receiving end of proton shuttles from the phosphorylated substrate could be understood as supplying electrons for the formation of the P_γ_–O_nuc_ bond. Concentrating these electron flows would be assisted by the low-dielectric, electrically insulating environment of the protein cavity.

Our current approach of using a low-dielectric environment to study phosphoryl transfers should enable rapid investigation of phosphoryl transfers in proteins to biologically relevant substrates such as water, sugars, metabolites, and certain alcohols and phenols. The theoretical principles of catalyzing phosphoryl transfer could be tested *in vitro* through their implementation in the design of *de novo* proteins and subsequent experimental characterization of their catalytic abilities. The biochemical significance of this body of work lies in understanding the catalytic chemical effects of protein surface characteristics in phosphatase and kinase active sites.^18,19^ For example, the results of the present work suggest that one could enhance kinase catalytic behavior by lowering the active site dielectric and excluding amino acids that sterically clash with the reacting chemical groups.

## Supplementary Material



## Notes

Mechanistic characterization in the β-γ Mg^2+^ binding mode; attempt to model the aqueous hydrolysis end state; convergence thresholds; and atomic cartesian coordinates and absolute energies (PDF)
