# Supplementary material for: Ab Initio Exploration of the Phosphoryl Transfer Reaction Provides Insights into Interpreting Models of SN2 Mechanisms
Source: J Phys Chem C Nanomater Interfaces. 2025 May 14;129(21):9686–98. doi: 10.1021/acs.jpcc.5c00499 (PMC12128102; doi:10.1021/acs.jpcc.5c00499)
Supplement: Supplementary file 1 [file jp5c00499_si_001.pdf]

SUPPORTING INFORMATION

"Ab Initio Exploration of the Phosphoryl Transfer Reaction Provides Insights into Interpreting Models of SN2 Mechanisms"

Robert F. Spaine, Fei Wang, Kenneth W. Foreman, Lee A. Solomon

Mechanistic Characterization in the β-γ Mg^{2+} Binding Mode

Table S1. Interatomic distances (Å) relevant to aqueous phosphoryl transfer to water from Mg·$MeTP^{2-}$ with magnesium bound in the β-γ Mg^{2+} binding mode. Ɛ=78.3550

State	Interatomic Distance (Å)	
	$P_γ$—O_l	$P_γ$—O_{nuc}
Reactants	1.68	3.57
Transition State	2.44	2.12
Products	2.93	1.87

Table S2. Mayer bond orders relevant to aqueous phosphoryl transfer to water from Mg·$MeTP^{2-}$ with magnesium bound in the β-γ Mg^{2+} binding mode. Ɛ=78.3550

State	Mayer Bond Order	
	$P_γ$—O_l	$P_γ$—O_{nuc}
Reactants	1.03	0.04
Transition State	0.22	0.49
Products	0.09	0.61

When magnesium is bound to $MeTP^{4-}$ in the β-γ bidentate configuration, most of the spatial association between $P_γ$ and O_{nuc} and spatial dissociation of $P_γ$ and O_l happens before the formation of the transition state (Table S1). Bond orders also imply that cleavage of the $P_γ$—O_l bond occurs mostly before the formation of the transition state (Table S2). In fact, this apparent $P_γ$—O_l bond cleavage is even more dramatic in the β-γ mode than the α-β-γ mode because the initial $P_γ$—O_l bond order is greater, at 1.03, and decreases to 0.23 at the transition state. In contrast, in the α-β-γ mode, the $P_γ$—O_l bond order decreases from 0.71 to 0.27 between the reactants and transition state (Table 4). A greater $P_γ$—O_l bond integrity in the β-γ mode reactant state may have contributed to the higher enthalpic barrier when magnesium is bound in the β-γ mode, compared to the α-β-γ mode (Table 1). In contrast to hydrolysis in the α-β-γ mode, in the β-γ mode, bond orders suggest more $P_γ$—O_{nuc} bond formation before the formation of the transition state (Tables 4 and S2). This stronger $P_γ$—O_{nuc} electronic association could be necessary to break the stronger $P_γ$—O_l bond of Mg·$MeTP^{2-}$ in the β-γ magnesium binding mode. In the β-γ mode, the sum of $P_γ$—O_l and $P_γ$—O_{nuc} bond orders is 0.71 at the transition state; but 1.07 and 0.70, for the reactants and products. The imaginary vibrational mode of the transition state shows a single progression from methyl triphosphate through the transition state to methyl diphosphate and a nascent cleaved phosphate. In summary, this imaginary vibrational mode, bond orders, and bond distances suggest simultaneous bond formation and bond breaking over the reaction coordinate. Therefore, we conclude a concerted hydrolysis mechanism for Mg·$MeTP^{2-}$ in the β-γ mode.

Attempt to Model the Aqueous Hydrolysis End State

We sought a more stable end state readily accessible from the metastable product state of aqueous Mg·$MeTP^{2-}$ hydrolysis. We attempted to model the end state of Mg·$MeTP^{2-}$

hydrolysis by first using Avogadro 1.2.0 to model proton transfer from the attacking water to the nearest (assisting) water oxygen atom, and the continued formation of the P_γ—O_{nuc} bond. Then we used Avogadro, with a UFF forcefield,[1] to perform a constrained optimization. This constrained optimization allowed the movement of O_{nuc}, P_γ, and the proton that transfers from O_{nuc} to the oxygen atom of the assisting water molecule. This was followed by unconstrained geometry optimization in ORCA 6.0.0. For the α-β-γ mode, this resulted in the formation of a hydronium ion from the assisting water and the transferred proton. For the β-γ mode, the proton transfer from the attacking to the assisting water was followed by the transfer of a different proton from the assisting water to one of the oxygen atoms on the cleaved phosphate. However, thermochemical calculations on these end states implied that $Mg\cdot MeTP^{2-}$ hydrolysis is endergonic by 27.0 $kcal\cdot mol^{-1}$ for the α-β-γ mode and 10.4 $kcal\cdot mol^{-1}$ for the β-γ mode relative to the respective $Mg\cdot MeTP^{2-}$ reactant states. Both of these results contradict the exergonic Gibbs free energy change described using *in vitro* data.[2] Moreover, for the α-β-γ mode, the end state we arrived at even had a higher Gibbs free energy than the metastable intermediate that preceded it by 2.2 $kcal\cdot mol^{-1}$, but a more favorable enthalpy by -1.1 $kcal\cdot mol^{-1}$. Accurately modeling the end state of MeTP, ATP, or GTP hydrolysis may require an approach that fully accounts for the solvation of the product species, as this solvation may be necessary to decrease electrostatic repulsion between the product species enough to reach an equilibrium state.[3,4] To characterize the full dissociation of product species within reasonable computational expense, we suggest using simulations that handle most of the explicit solvent using classical mechanics (as in QM/MM) or a computationally inexpensive level of QM theory.[3,4]

Convergence Thresholds

Table S3. Convergence thresholds for geometry optimizations in ORCA 6.0.0.

Convergence Parameter	ORCA Keyword					
	looseopt	opt normalopt	tightopt	verytightopt	OptTS (first round)	OptTS (final round)
Energy Change (Eh)	3.0000e-05	5.0000e-06	1.0000e-06	2.0000e-07	5.0000e-06	2.0000e-07
Max. Gradient (Eh/Bohr)	2.0000e-03	3.0000e-04	1.0000e-04	3.0000e-05	3.0000e-04	3.0000e-05
RMS Gradient (Eh/Bohr)	5.0000e-04	1.0000e-04	3.0000e-05	8.0000e-06	1.0000e-04	8.0000e-06
Max. Displacement (Bohr)	1.0000e-02	4.0000e-03	1.0000e-03	2.0000e-04	4.0000e-03	2.0000e-04
RMS Displacement (Bohr)	7.0000e-03	2.0000e-03	6.0000e-04	1.0000e-04	2.0000e-03	1.0000e-04
Strict Convergence	false	false	false	false	false	false

Table S4. Convergence thresholds for NEB-CI calculations in ORCA 6.0.0.

Convergence Parameter	ORCA Keyword	
	NEB-CI	
	Regular Images	Climbing Image
Max(|Fp|) (Eh/Bohr)	5.00e-03	N/A
RMS(Fp) (Eh/Bohr)	2.50e-03	N/A
Max(|F|) (Eh/Bohr)	N/A	5.00e-04
RMS(F) (Eh/Bohr)	N/A	2.50e-04

Atomic Cartesian Coordinates and Absolute Energies

The absolute energy of each system (in Hartrees) is provided directly above its atomic coordinates.

Mg·MeTP^{2-} Hydrolysis Within an Explicit Water Shell in Implicit Water, α-β-γ Mode

Aqueous Mg·MeTP^{2-} Hydrolysis Reactants: α-β-γ Mode, E -3394.563470485054

P	-2.00277808361840	0.76996003940340	-0.11713092974131
O	-0.40440017842942	1.24831673288687	0.07336442779337
O	-2.67821072020629	1.24062235261600	1.16467583106385
O	-1.93780484980476	-0.76735248768136	-0.23391789392968
O	-2.48559017088989	1.45993413273048	-1.38890492072769
P	0.74113442066167	1.02666055568294	-1.04324861840938
O	-5.47610684596843	1.08051492851377	0.75897253373601
H	-4.10934680145836	1.12421519360562	-1.92739962414614
H	-4.52852699802409	1.15316252985007	0.97629479251005
H	-1.07083269647836	3.68768795638532	2.19919933513467
O	-1.62287726141329	3.17064437612491	2.81535632058510
H	-2.06906768324266	2.52731582142879	2.22853050840136
H	-1.83512968943491	-2.33844349248985	2.34555543325167
O	-2.64042310210168	-2.49119721616165	1.81400518243846
H	-2.58986883651595	-1.77365982254539	1.15288426668049
H	-0.32776129541073	1.75980837089412	3.13244578670904
O	0.30775433345256	1.04455777404721	2.95177230866326
H	0.30595959235453	1.00396997546783	1.98512548006758
H	0.74040608337094	3.91217432106147	0.80199036325364
O	-0.03860740876947	4.47801869413765	0.86045332261373
H	-0.51765959646972	4.31059469031095	0.02620791896013
H	-3.00034975953969	0.39827996185716	2.70265774213922
O	-3.25468650825833	-0.04016613560347	3.53896476018338
H	-3.26179227523451	-0.97538607317564	3.30019588132032

H	0.22967196178939	-1.86018741713918	2.13460265212486
O	-0.17902041440081	-1.79045513824628	3.01597821252305
H	-0.16919524327085	-0.83090873958628	3.17844640648171
H	-4.55305229868048	-1.02601618210297	-1.96714731547436
O	-4.08773116300445	-1.83134992471526	-1.68284501795450
H	-3.44746084253262	-1.49882510651406	-1.02555303837214
O	1.98283920747268	0.54111230039816	-0.08908026189360
O	0.35073000632327	-0.13646321399406	-1.92777440014012
O	1.14128650699159	2.32689664841153	-1.66722134714944
P	2.23543630432624	-1.02438330139091	0.26182531849343
O	2.91349119894448	-0.81259778475198	1.69955354245119
O	0.88853853788746	-1.72327265567599	0.41882014953355
O	3.17302493511471	-1.64556490160904	-0.73885975850208
C	3.43995645037989	-1.97940874896133	2.38391250821440
H	3.81522689989760	-1.62195903746130	3.34126431608392
H	2.64450117050880	-2.71002268160427	2.54461021194481
H	4.25209845182647	-2.41658445163327	1.79929545317255
H	4.38898141814379	0.39936820596193	1.68675010403801
O	5.23782243897253	0.86381668469182	1.61442508139818
H	3.62110363248995	-0.54818163720732	-2.10512394754950
O	3.92248345734957	0.10361955158647	-2.76774780329478
H	3.11204773636400	0.48888132188139	-3.11930920280439
H	-0.34915599683684	3.52726217164824	-1.88081751485893
O	-1.21600635678935	3.92530422528970	-1.69291500653020
H	-1.78935429961446	3.13877324531117	-1.59343887549563
H	2.38557012435186	3.38123625960281	-0.82050143740264
O	3.14396053028810	3.83659106297321	-0.41478648215321
H	3.88576903500531	3.22760656985015	-0.59318182832087
O	-5.03202443193965	0.82737913006958	-2.09915374086136
H	-5.48425455766500	1.02765320357633	-0.21011505440733
H	-5.33961667172926	1.33800967931320	-2.85533954162941
Mg	-0.49679122004100	-1.89773782416323	-1.17664268566374
O	1.06216027000606	-3.00801700251270	-2.16104147672917
H	1.04687174239976	-2.90888333082708	-3.12027428900056
H	1.92922850395478	-2.66697942816255	-1.87581379671642
O	-1.64577208542701	-2.12629077001703	-2.93870010826444
H	-1.50708132225540	-2.95662499065589	-3.40838663867233
H	-2.59640499579211	-2.10241376459252	-2.70076342119760
O	-1.28519290084475	-3.68983114303876	-0.35820348693882
H	-1.83390291962067	-3.45189325994964	0.41505953483826
H	-0.55123126701114	-4.21068711224383	0.01768991730088
H	1.17182962278252	-3.56005699726567	0.78298649864312
O	1.11587390321817	-4.53000638817745	0.83953886237414
H	1.06126507797964	-4.71412565952824	1.78410295254989
H	4.72478885264111	1.31585840086012	-1.58549499416275
O	5.16128382012531	1.94687485656167	-0.98351452145662
H	5.20328568540694	1.49684901854749	-0.11914315432261
H	5.89819683594302	0.17326387784603	1.74186221720234

Aqueous Mg·MeTP^{2-} Hydrolysis Transition State: α-β-γ Mode, E -3394.520246196900

P	-2.40867953399624	0.97666618877030	-0.04748575207883
O	-0.15084530028566	1.57235118133545	0.26596186584384
O	-2.63214826063497	1.47301506495541	1.35508891269677
O	-1.97933256934404	-0.47120129946736	-0.24898427338445
O	-2.48148228990959	1.88417407234252	-1.24065344419769
P	0.86789822928945	1.37789677690782	-0.86830451716157
O	-4.45892240417581	0.42101535830889	-0.15503204165809
H	-4.51678902134342	0.94094266616569	-3.25693015382412
H	-4.96531983858345	1.04896899730564	0.37610592618847
H	-1.18993300062398	3.80193069761023	2.68141054393123
O	-1.70656483266655	3.23222651340377	3.27645584094220
H	-2.12267722813145	2.61128632575979	2.64950407706522
H	-1.86355122877900	-2.09764513913350	2.29761303051157
O	-2.66836199317478	-2.25869461494805	1.76509302674011
H	-2.60889068720542	-1.55734982849116	1.08855155047491
H	-0.25744336874887	1.82165544304686	3.30293686933688
O	0.36158945235845	1.15712895121326	2.96117885005975
H	0.25706427384455	1.23655897921993	1.99126093310262
H	-0.10762464016604	3.27046808360303	0.86696967551071
O	-0.15688195201698	4.22061360364078	1.10013781784027
H	-0.52940099116462	4.60849333235633	0.29547330028916
H	-3.60633648317971	0.51448587146007	2.56171862035950
O	-4.06833823812017	-0.09307693628843	3.16964836977030
H	-3.76564976125789	-0.96202677559669	2.86436267448486
H	0.23476027189006	-1.67390679854422	2.07879364027543
O	-0.19999091873165	-1.61316805424101	2.94908946156564
H	-0.13422017725945	-0.65863295330082	3.13927775077360
H	-4.44662877220212	-1.38059273134353	-2.27543757392764
O	-3.91477011286743	-2.09780747390445	-1.89404980929391
H	-3.49187712567947	-1.68779045546447	-1.12406990984792
O	2.22889316590711	0.74725788588275	-0.10498041871007
O	0.45349522267082	0.27998320015700	-1.85858779165776
O	1.35616668279604	2.66171923555986	-1.51056731825692
P	2.35543911085543	-0.80802563756074	0.26758684951189
O	3.04429067793161	-0.64723575355182	1.71492599185783
O	0.96036471414395	-1.42004649459759	0.41729365400730
O	3.26166848997425	-1.54207883608995	-0.69090650594362
C	3.47447297838060	-1.84946007982563	2.39863450686765
H	3.94153077101336	-1.52018348990269	3.32568154438235
H	2.61289001627838	-2.48281474668001	2.62096946115214
H	4.19951182095729	-2.39100025840239	1.78663254853717
H	4.73855581708068	0.32328091075666	1.32231563870550
O	5.57497644852957	0.60662889929223	0.92387134387443
H	3.27072531865185	-0.76306283123434	-2.41856074533698
O	3.05629596237658	-0.16239578101162	-3.15376696788919
H	2.12964366452214	0.06158754094832	-2.96556148398555

H	-0.12823656094843	3.80802693950616	-1.75973150061618
O	-1.01320074749728	4.21654410176608	-1.76245281212234
H	-1.59574756265499	3.46236494850800	-1.55248943365410
H	2.88639930169774	3.43824741407639	-1.03978957032235
O	3.78696663256652	3.77783938708039	-0.86719202312842
H	4.35201595207250	3.04605207822680	-1.17213988912075
O	-5.12380200649582	0.35209144790550	-2.79201188188672
H	-4.80099689882857	0.49071734646871	-1.07526307110047
H	-6.00397818658151	0.57840129166921	-3.11440928874651
Mg	-0.41789626197314	-1.46300736641353	-1.18918927055137
O	1.09637770686877	-2.66143628169046	-2.19035678214087
H	1.20664171942215	-2.39213591054408	-3.11012687834075
H	1.95996985547574	-2.48444991113169	-1.77464814693103
O	-1.48159029252966	-1.63060864464248	-3.02150256696984
H	-1.14593902918692	-2.29111348660394	-3.63790466383811
H	-2.40125887577751	-1.89863036819231	-2.80784440506270
O	-1.22220220035190	-3.30883823291468	-0.47523044045407
H	-1.79367147181735	-3.15217228264146	0.29842335151883
H	-0.49430550449510	-3.85858741182531	-0.13090229219279
H	1.21727069710400	-3.27783744006192	0.69119582383277
O	1.15945674189354	-4.24966413171846	0.69543739457945
H	1.08729558601845	-4.48362724642996	1.62761758086195
H	4.40768655090872	0.97106374885942	-2.18953960419276
O	5.14756524467944	1.43145094525955	-1.76190175684932
H	5.22606251574264	1.03787552978091	-0.87343414860639
H	6.14154473548463	-0.17070527471892	0.98472070652989

Aqueous Mg·MeTP^{2-} Hydrolysis Products: α-β-γ Mode, E -3394.524646024333

P	-2.76686664789256	0.84286751882848	-0.17933301366152
O	0.06789283024387	1.65865945869579	0.48869872093292
O	-3.02406675244869	1.55050343440476	1.13097303446087
O	-2.08509917836360	-0.51930643269648	-0.08362525277770
O	-2.49636443106991	1.66045725694839	-1.41389868641256
P	0.98520819159818	1.33539562200848	-0.68355382246710
O	-4.47571958987944	0.25692079594477	-0.48833430462895
H	-4.14854628659641	0.01457095456807	-3.45643333095953
H	-5.13733204449577	0.86366180083654	-0.12118864752294
H	-1.43846116671082	3.79837734683058	2.47653166500093
O	-2.00665977412375	3.25403769205488	3.04918326848805
H	-2.41676542525918	2.63953752900906	2.41092270655115
H	-1.89502769806792	-2.01856792258125	2.54238229960502
O	-2.70957748931530	-2.16613084977136	2.02066153352634
H	-2.61696801714067	-1.51088945529966	1.29878687937853
H	-0.61783360735228	1.86280425280822	3.36599497077497
O	0.08243148565716	1.21863026446718	3.16993446027073
H	0.17119062425164	1.28925694933056	2.19481125361287
H	-0.06978268009843	3.35124570334693	0.88070894416575
O	-0.26270307396282	4.30366077664365	1.03329708470203

H	-0.63752196528808	4.57114674000606	0.18186252720603
H	-3.90700357393348	0.61591831826115	2.43316939007695
O	-4.32185277307232	0.01781024160156	3.08337839147323
H	-3.89454501344493	-0.83235995970278	2.89203700646103
H	0.23719167372849	-1.64206274128141	2.32589432155987
O	-0.22116690351868	-1.56546187487975	3.18248521435114
H	-0.22027641230369	-0.59977729228938	3.33080303394225
H	-4.56161865057225	-2.09220378636363	-2.01557777017637
O	-3.91961295468047	-2.68154972867069	-1.59648575678768
H	-3.65464549022495	-2.21439074869510	-0.79224991057056
O	2.35286758172456	0.62988094963348	0.01840617571290
O	0.45972752618423	0.22829557789246	-1.61661086464304
O	1.52336235590465	2.54865457214631	-1.42698105664132
P	2.38995990260746	-0.91313381714212	0.44195346045210
O	3.12642901518202	-0.75153783942355	1.86755064392992
O	0.96782456296253	-1.44296517287922	0.64932373797354
O	3.22679567205448	-1.73217298003981	-0.51301142868257
C	3.47241942822948	-1.95437881411273	2.59497348481479
H	4.03221796852250	-1.63005776310690	3.47106761386499
H	2.56477871911332	-2.47608760969009	2.90627327204197
H	4.09385982978750	-2.60553108004568	1.97557705228622
H	4.88199762393091	0.08281800739416	1.39277589370340
O	5.73424166661882	0.21045283678412	0.94967955922394
H	3.21056204627684	-0.96839975402805	-2.26658250301441
O	2.99144893919660	-0.36679411807465	-2.99910027585713
H	2.08692346824682	-0.09325011526365	-2.77073328237356
H	-0.05261709397365	3.59146043777652	-1.79611082613216
O	-0.95223458899391	3.95838310163701	-1.86569167226016
H	-1.52559760641180	3.19064313618802	-1.67825805677546
H	3.09520036808888	3.25496403250467	-1.01641371318125
O	4.01743281871014	3.54834384584374	-0.86939633864786
H	4.53555768056947	2.77382179267482	-1.15136753018597
O	-4.91919240474080	-0.26140464669454	-2.94279718001125
H	-4.68876605556525	0.10196840544473	-1.47675247417397
H	-5.67215882851725	0.20174883523933	-3.33048213055006
Mg	-0.46227310524020	-1.47042865755819	-0.92838066330254
O	0.98445486662528	-2.74045187283584	-1.95736481418308
H	1.10052455437803	-2.45654007741844	-2.87211993058441
H	1.85848334689514	-2.60724741406444	-1.54592743386293
O	-1.60585643419806	-1.65439307157280	-2.72400065712661
H	-1.21740079423424	-2.19707314968403	-3.41952548515537
H	-2.44920116952230	-2.09139009200066	-2.48295317131768
O	-1.24295314600730	-3.31672864805732	-0.17183875421249
H	-1.83316740707442	-3.15430431821293	0.58484947337166
H	-0.51914600545356	-3.85850733961134	0.19311569942944
H	1.18929131238612	-3.29956141853485	0.97415216925588
O	1.12092081544439	-4.27042517920951	0.99767696515930
H	1.05786537599516	-4.48595584173434	1.93499369043303

H	4.45991909360919	0.69484161713615	-2.10049726321246
O	5.24498404762284	1.11024828861596	-1.70954959130580
H	5.33654228591995	0.71655845434855	-0.82234421041386
H	6.10615156148252	-0.67817296462780	0.90556920557858

Mg·MeTP^{2-} Hydrolysis Within an Explicit Water Shell in Implicit Water, β-γ Mode

Aqueous Mg·MeTP^{2-} Hydrolysis Reactants: β-γ Mode, E -3394.561595406278

P	-1.50224968842176	-0.82808745294535	0.48184453029037
O	0.12406485078180	-1.08438717610455	0.82553622765589
O	-1.99877027839745	-2.21487483864149	0.08588216826956
O	-1.47128521217873	0.16882643406418	-0.69540723236084
O	-2.10333742184430	-0.26498947905739	1.76345148508928
P	1.12626563436785	0.08775197052734	1.28913719180691
O	-4.82867737337899	-2.09247547842322	0.20911552247065
H	-3.75561845326284	-0.14282429440787	2.14549606937678
H	-3.86354003743593	-2.23590874060341	0.19115026738269
H	0.83204268384001	-3.73140387593979	-0.44494090492505
O	-0.02539497752042	-3.93540864282359	-0.86426458598020
H	-0.68943284377300	-3.39426849060106	-0.39624017199049
H	-4.26064076281422	-0.14963991946528	-2.37439622168726
O	-5.04412452048587	-0.10884568118525	-1.80436375308771
H	-4.95542059343229	-0.86060646518493	-1.18652239639837
H	0.10774959746381	-2.49053221786962	-2.07350272512633
O	0.26464248192545	-1.66160476232896	-2.56465924434077
H	-0.09822335364408	-0.97332950573922	-1.99004052576089
H	0.37391052542841	-2.94280964105800	1.84390967769890
O	0.20220800164518	-3.40858585600994	2.67280535910931
H	-0.16294336689096	-2.70825331991541	3.24043753103937
H	-2.55851757309687	-2.64546081525532	-1.59374618465750
O	-2.92607842002511	-2.92897035356398	-2.45303767374136
H	-3.03264493702844	-2.10406068027372	-2.93987461174153
H	-2.11888421569886	0.00748400832520	-2.28819223895949
O	-2.52454692451552	0.18163944513918	-3.16766466327791
H	-1.95480518084815	-0.26129003708556	-3.80667912799892
H	-4.78965010989824	1.30987664582266	0.94657882808245
O	-4.62636056856486	1.85702154112105	0.15828692408131
H	-4.72807310597909	1.23068100386254	-0.58653588313097
O	2.32394131099738	-0.18877703086306	0.23352592396419
O	0.49509766443493	1.43200926743025	0.99248608428553
O	1.62613397477571	-0.16784419337499	2.68410669166367
P	3.75304422019589	0.58827332103782	0.08850419678212
O	3.56670014083103	0.91426962634653	-1.50001410452279
O	3.77243268687677	1.86033189372363	0.88925880163963
O	4.84972628942382	-0.42001915349545	0.29478305301504
C	4.59334066104330	1.67576259172679	-2.17282432175129
H	4.28045174798603	1.76080117182680	-3.21270779471482
H	4.67696572892495	2.66898795310103	-1.72540288373853

H	5.54932170816883	1.14933577383999	-2.11478239361025
H	1.77083243040658	1.26654002193597	-1.90466960873187
O	0.86023557691033	1.53676795262490	-2.11588430237964
H	0.58310553286839	0.98557227101429	-2.85593912750784
H	2.65917052770761	3.18596294187192	1.42063350885824
O	1.90543904721382	3.70605744143639	1.76170573588860
H	1.24762206087013	3.00488285019186	1.89667090231003
H	0.12386050851441	-0.67238879086559	3.82548777941844
O	-0.74528265566290	-1.02955337197709	4.07393165147252
H	-1.31108084035489	-0.76902207704949	3.31901025887090
H	4.23803942588327	-1.61051421396286	1.69380700050181
O	3.69366027756214	-2.15305637081077	2.29115672970850
H	2.98607495015857	-1.54677692882895	2.57128967039861
H	2.90208819013393	-2.95689331003272	0.72341884445935
O	2.62010822789778	-3.38728601559615	-0.10279532850619
H	2.85239026626008	-2.76440428995546	-0.81376941572820
H	3.35652452745012	-0.89113228750568	-2.30266298545277
O	3.04403055423891	-1.78580881240218	-2.49873089302674
H	2.07274393603146	-1.68453429330063	-2.55911259860305
O	-4.73520961059540	-0.06736314986438	2.23281887916233
H	-4.95944491173020	-1.49648912332802	0.96578618314227
H	-4.92098378731114	-0.05179266492784	3.17742117586694
Mg	-0.59712018592097	2.03730429938399	-0.64459442949967
O	0.34799544993578	3.94704032887805	-0.54549028863691
H	-0.24997719608924	4.70168126777072	-0.50471167586005
H	0.93891429169435	4.02658414446621	0.23181357028222
O	-2.12159101244805	2.81394118503218	0.62006608132289
H	-2.20146860530783	3.77362374219357	0.58187931388620
H	-3.02188531493511	2.46196818133684	0.42296109084036
O	-1.75531994308937	2.72547354758400	-2.31157568138132
H	-2.54423151349729	3.19406325651188	-2.01400670861466
H	-2.08778319477013	1.95510372249668	-2.81248722266126

Aqueous Mg·MeTP^{2-} Hydrolysis Transition State: β-γ Mode, E -3394.515532460162

P	-1.91798274016624	-0.93687211317531	0.58148769002547
O	0.52277451639657	-1.02802456091818	0.66420822532950
O	-1.79116754422789	-2.31309153787892	-0.00707229491422
O	-1.78056489807036	0.27749777449930	-0.31493959896581
O	-1.98774273118862	-0.71519920346221	2.06760111828386
P	1.29745627607616	0.16373443307949	1.23759025989935
O	-4.01628026280494	-0.97500501291528	0.31268113446338
H	-3.55017752656039	0.08314965241121	2.73269901192958
H	-4.30231810070124	-1.89808200566069	0.26303921301663
H	1.14386714015591	-3.88303297106801	-0.33945784004926
O	0.26562939146648	-4.18008435527961	-0.64274563088222
H	-0.36810640937822	-3.54528726755455	-0.26686322502606
H	-3.51015953105885	-0.14593669735192	-2.88134674651093
O	-4.40447600623697	-0.21045558498659	-2.51391892222328

H	-4.26426967262654	-0.28027383627675	-1.55740505324543
H	0.51931013402028	-2.60866197191515	-1.98880429487117
O	0.67609943224247	-1.65755651308382	-2.08426626208247
H	0.61600802453737	-1.33539068325856	-1.16046027748945
H	0.54543459171288	-2.50866812796178	1.76236610449522
O	0.51157765336499	-3.19055920659664	2.46126076422004
H	0.31063937759182	-2.67161527722015	3.25269730854967
H	-2.83039587775052	-2.82585330430950	-1.40362016981000
O	-3.42769841928427	-3.11023799882301	-2.12159263745528
H	-3.87264724973132	-2.29387780659264	-2.38631202952480
H	-1.65776825675739	0.01921761295784	-2.04671585452744
O	-1.48844392864193	-0.09895603886840	-3.00470329188863
H	-0.82655422706558	-0.81155911248865	-2.99777833543457
H	-4.88772496785508	1.60799066241458	1.27916007919191
O	-4.80122731317660	2.21550247799203	0.52464997252292
H	-4.76374044148792	1.63892145108912	-0.24795377938159
O	2.48266922809528	0.38260473365036	0.08473642215578
O	0.47332513299354	1.46093861162588	1.22647704782680
O	1.99229249770431	-0.08031936541075	2.56977343416342
P	3.97616640707313	1.00185436827425	0.16413896648737
O	4.20225663249850	1.00360630515755	-1.45325331027303
O	3.96567265634332	2.41828583754717	0.68412848087309
O	4.93367865219214	0.02710142942428	0.80614774455107
C	5.49823124149114	1.40212898962455	-1.94257938833831
H	5.44668697113821	1.35736356173561	-3.03009775807789
H	5.72536262376848	2.42428785582048	-1.62755920468137
H	6.26877140124551	0.71619059963224	-1.58068975914781
H	1.34843379612505	1.08133410338676	-1.45758176638627
O	0.58429859471539	1.58446725166178	-1.77674365391905
H	0.13805799602292	1.01548848095839	-2.42329683632496
H	2.68227266951006	3.37486562671422	1.46919769378249
O	1.86595380174484	3.71460587780393	1.88804725776554
H	1.31249433796854	2.90836848100463	1.90108324096079
H	0.64540138621119	-0.55669592431598	3.78781211691485
O	-0.17254827353208	-0.87222136556953	4.21597546631521
H	-0.83552826053974	-0.80612681935198	3.50508174450365
H	4.38636916545145	-1.43071572916110	1.70469244261286
O	3.86293515505145	-2.08388713233952	2.21066330581347
H	3.10583087666401	-1.53291589495283	2.49101276529066
H	3.21172297238258	-2.93881032884965	0.71716457319825
O	2.94919436454413	-3.37650679889871	-0.11684150847318
H	3.18725576825261	-2.74430482462626	-0.81594532920759
H	3.73181618118057	-0.72198721008330	-2.12398158275939
O	3.44752788236199	-1.60755644006932	-2.40138027503294
H	2.47103376902266	-1.56343215665270	-2.38436986503842
O	-4.48024657973722	0.37127244311046	2.65948050375027
H	-4.45420964433182	-0.58722363897126	1.10086259460311
H	-4.95368608042786	-0.05315165586775	3.38309836242726

Mg	-0.79796163421831	2.09451982103044	-0.21205093420623
O	0.10895727362514	4.04032386039958	-0.23053427937115
H	-0.49938091181783	4.77453373480728	-0.09071209645604
H	0.78066586943171	4.11011460243268	0.48079725742291
O	-2.20885047037722	2.83378369045206	1.17198626441714
H	-2.14662989037390	3.77981233976189	1.34492212768701
H	-3.14177513595591	2.66793359844126	0.91148179862389
O	-2.08651169405589	2.76278363889099	-1.80552260622065
H	-3.01295270256207	2.62399803925460	-1.57073329394945
H	-1.95440445967405	2.24155452572010	-2.60837280192811

Aqueous Mg·MeTP^{2-} Hydrolysis Products: β-γ Mode, E -3394.517453966906

P	-2.43557050304304	-0.78040077964145	0.46261652001734
O	0.47721321379724	-1.08807698103636	0.59890222737008
O	-2.20842477988628	-2.21398157153753	0.05419276176722
O	-2.03872579621118	0.30107369070058	-0.53251881781772
O	-2.37986217932446	-0.39639813474545	1.92088633430633
P	1.19179817100244	0.16652406773537	1.08201461370412
O	-4.28259043216777	-0.71850188581340	0.18915522839720
H	-4.10060293581300	0.42005042587464	2.63058132372419
H	-4.66352469274516	-1.60233785368065	0.06378853461690
H	0.66886803333692	-3.99342226291802	-0.03265570945142
O	-0.22877474808262	-4.23731623506312	-0.32324004363720
H	-0.80841397013882	-3.50980465024381	-0.03818666967493
H	-3.52778041401914	-0.73181428003299	-3.18805986519241
O	-4.47894760121354	-0.79107380187567	-2.98777191037320
H	-4.56294267859411	-0.35820896804240	-2.12901422859473
H	0.21375835532107	-2.87052715151386	-1.83574195056849
O	0.43946871908801	-1.94631847258022	-2.01831386742438
H	0.42747186356757	-1.54015423387330	-1.12130798068369
H	0.26116964521335	-2.36837929469615	1.86582544425192
O	0.10946812445831	-2.93669845847411	2.64828523493737
H	-0.11764614289609	-2.29348949156897	3.33470441346543
H	-3.28804605149500	-3.06354463279566	-1.14666839759404
O	-3.94890365179857	-3.40424457877540	-1.77842397848633
H	-4.22968517199215	-2.60620537374454	-2.25514968118366
H	-1.84964326753658	-0.12923118386460	-2.23259128855027
O	-1.63935253508488	-0.44486711249645	-3.13635823042732
H	-0.99073328940978	-1.14910034706238	-2.95254710581403
H	-5.10930884974323	1.95069477329107	0.86540441525956
O	-4.85052323602734	2.44783497983380	0.07369837969859
H	-4.76208005628082	1.77291726877913	-0.61065443525638
O	2.38313839888752	0.36649823734745	-0.07525242544616
O	0.34124638725179	1.44571575906068	0.96337777460620
O	1.86863753968300	0.05554811380938	2.44357043214136
P	3.93131577589283	0.81507848491793	-0.02950860051665
O	4.18227908394821	0.58285529121311	-1.62710541579630
O	4.06445217670003	2.28200855741379	0.30717516597854

O	4.77195764153751	-0.16055898508751	0.75871869226784
C	5.51734486728925	0.79061768412255	-2.12775879929822
H	5.47962862652317	0.60228582026827	-3.20044296037913
H	5.83567835463968	1.82094994378121	-1.94655984669324
H	6.21419306008869	0.09299087792926	-1.65538191299610
H	1.25908183243222	0.87934896199456	-1.66415630728938
O	0.48107991844711	1.32827757929549	-2.02910938663581
H	0.05235221193837	0.69410970023820	-2.62270189344449
H	2.71866819189166	3.26840379071589	0.93886688989871
O	1.90132143029016	3.67212199422690	1.29625025825192
H	1.31300164807655	2.89557804176104	1.39241258434867
H	0.34573622492349	-0.15448047257641	3.59690790730854
O	-0.51330637171180	-0.35863811190370	4.00894332977764
H	-1.14676885604612	-0.33999970934488	3.26663007313050
H	4.12315749417671	-1.55781432073840	1.70606338011833
O	3.55923481465346	-2.13095073907477	2.26233171761672
H	2.85002953108067	-1.50300482060098	2.50325828120949
H	2.85170815386190	-3.17954349471370	0.91506709227635
O	2.56503069624136	-3.70184817118718	0.13983463296362
H	2.84709061468376	-3.16910247632885	-0.62281822781430
H	3.56047693018225	-1.20324361963588	-2.05488620092470
O	3.21499704231490	-2.09168361327412	-2.23470447750215
H	2.24444614752284	-1.97744018423965	-2.25874044700944
O	-4.99704429009499	0.59692374964415	2.29520741143556
H	-4.74412248133420	-0.27715570299715	0.97023050211313
H	-5.60937340834731	0.21240420718739	2.93336809801620
Mg	-0.89991708813760	2.01640836040699	-0.52627431903461
O	0.06080993098110	3.92747205874683	-0.74057382361679
H	-0.51734353852249	4.68531009122212	-0.59895988288059
H	0.78339077688324	4.01567856108946	-0.08239769970073
O	-2.25738803353700	2.92805925209040	0.82590567298725
H	-2.13543170980628	3.87640335633125	0.94642269818754
H	-3.19395895240900	2.81020020428655	0.55277651612375
O	-2.16246978745712	2.64595657480165	-2.16590401392116
H	-2.95149481519504	2.08919062687401	-2.19306817907646
H	-1.74303231270575	2.52156407078860	-3.02636856156742

Constructs Related to Phosphorane and Metaphosphate Intermediates

Phosphorane-Like Structure from Constrained Optimization, E -3394.474245885435

P	-2.19176401287042	0.81471037878152	-0.17194240360047
O	-0.54975256761842	1.06906532581217	0.07605362772067
O	-2.49289977453838	1.40034729978356	1.25433440040581
O	-1.98448244475008	-0.76032240181119	-0.34410337805366
O	-2.20880706607990	1.68145915544193	-1.47631930887249
P	0.62458071696429	0.97885819149950	-0.97771289675174
O	-3.84437974249067	0.54492958247000	-0.30788191493163
H	-4.30373499147595	0.68172016034191	-3.34557189059885

H	-4.37552503377305	1.17291386830085	0.20251792774752
H	-0.99095770986690	3.82987620715607	2.31981586163647
O	-1.63892235384239	3.33614411912663	2.85332537633077
H	-1.96737274195873	2.64590976031042	2.22368569143722
H	-1.99068678034324	-2.07829069491148	2.39318655400546
O	-2.73735636540112	-2.24943577678184	1.78944068300525
H	-2.56027922922099	-1.62579767773469	1.04610900105665
H	-0.28681643463164	1.89929432584821	3.14488178954708
O	0.29565034866172	1.18036242371684	2.85041828280441
H	0.07362896629206	1.10267637922773	1.90690178022710
H	0.91342862132042	3.76107443834696	0.99001857564402
O	0.24223924377805	4.45172313590482	1.04519755865540
H	-0.28573731421049	4.33895521405196	0.22910339519885
H	-3.54775000107600	0.60274621648936	2.33683903348761
O	-4.11190028928776	0.06888103303542	2.94717973027695
H	-3.85764773656096	-0.83764931292221	2.71756164274517
H	0.11010890523874	-1.76149512542499	2.29849620899693
O	-0.32581210606957	-1.59507250403801	3.15342132017935
H	-0.23287359393886	-0.62996972629006	3.24613105336639
H	-4.43428159525129	-1.64899670926612	-2.10894854343487
O	-3.88619838313517	-2.29279501640850	-1.63854583916076
H	-3.31096688065917	-1.73409679136692	-1.06900171106977
O	1.89105562947973	0.56246391168095	0.01241172457919
O	0.41963202543688	-0.20874959001647	-1.91155298703903
O	1.02654244535438	2.28888038982070	-1.59201052118323
P	2.17143501926405	-0.96538784141692	0.45381853112101
O	2.80280363438436	-0.66305729641875	1.89966316046479
O	0.83256261257137	-1.68869648854844	0.59359305158164
O	3.16710012207298	-1.64117682507088	-0.45405683836649
C	3.32877417337119	-1.78018294085233	2.65764047266230
H	3.74196812450037	-1.35712758661233	3.57188662162762
H	2.52534810885235	-2.47917061268426	2.90030301969214
H	4.11429821306991	-2.28377905931294	2.08946195830882
H	4.48098241645998	0.42435135986393	1.47968486880890
O	5.34087077687905	0.58790028506422	1.06579200336885
H	3.28545926572631	-0.96572023381182	-2.17205073781173
O	3.12485987557489	-0.44465702775199	-2.98006316214286
H	2.16707007113731	-0.29710691955698	-2.93195805867397
H	-0.22598093214581	3.73929766934140	-1.68559670882223
O	-1.09241860852131	4.08624114286467	-1.41624600039864
H	-1.61035171479841	3.23840108217433	-1.38116392114995
H	2.36318417413001	3.28600973952707	-0.83747605719770
O	3.21790040437629	3.66245937452414	-0.55927485196095
H	3.84970837902764	3.00076263544999	-0.89466895142078
O	-4.86642016890736	0.23517247391545	-2.70060637297327
H	-4.23576055125454	0.46672019894946	-1.23017661837295
H	-5.74296567883309	0.62400631607175	-2.80825184034574
Mg	-0.47388882936014	-1.89839600296233	-1.07732231695897

O	1.13706023004079	-3.11732867527413	-1.87876361835828
H	1.22659685081897	-3.01012658418031	-2.83327680594355
H	1.97944932846713	-2.79824735118879	-1.50767220302088
O	-1.46844579217443	-2.31800651282737	-2.92012586614917
H	-1.18600106465429	-3.13153042219800	-3.35350510789602
H	-2.41025607889246	-2.45022950389554	-2.68052659556702
O	-1.28932648821747	-3.67128934405926	-0.19717547115362
H	-1.87686488214485	-3.39033068170705	0.52994661938612
H	-0.56975244307585	-4.15758477910962	0.24553651188882
H	1.12082855886911	-3.47211584092045	1.03156800853996
O	1.07485814904052	-4.43931258011731	1.14266045822427
H	0.99068640370905	-4.56983356604144	2.09379737046318
H	4.16482671634181	0.94533145229356	-2.02437287957922
O	4.79813754327384	1.52087647524339	-1.56389087609699
H	4.94785996554371	1.10197140309956	-0.69629759169790
H	5.73391936200201	-0.29047512203851	1.00070997156261

Reactant-Like State from Unconstrained Optimization of the Phosphorane-Like Structure, E -3394.563228841340

P	-1.98408253558832	0.72693511877540	-0.25945094275730
O	-0.40119928289637	1.21486465470145	0.01399707706073
O	-2.73973117504073	1.23143211241389	0.96328344457066
O	-1.90895287922752	-0.81283691490883	-0.32595453937510
O	-2.39098649069760	1.37588887458165	-1.57839005351095
P	0.80567278492595	0.97826228703852	-1.03145915678778
O	-5.46995763522129	0.68038521335593	0.40017219026070
H	-3.96661639380233	0.99021289540355	-2.22526831172748
H	-4.56234007952725	0.89986663941999	0.67813670985218
H	-1.19805365841317	3.75033152397960	1.94026280233465
O	-1.79611183613616	3.27106577180398	2.54380308288066
H	-2.20653783398384	2.59535787927408	1.96693122131723
H	-1.99687682018154	-2.25700468894429	2.32402406947074
O	-2.76079607421947	-2.45092047396036	1.74708570577878
H	-2.67903784868440	-1.76062730715677	1.06162509456864
H	-0.53931820343935	1.89991834955014	3.02767421861266
O	0.12068404553622	1.18604623818465	2.96021820499119
H	0.22742137967462	1.09051887573550	2.00398542784326
H	0.69709016507260	3.86051471357559	0.64954579207851
O	-0.05258580040495	4.46759285258742	0.65111991931563
H	-0.50435016612989	4.28523848285368	-0.19500749670487
H	-3.13120346691359	0.47271377074154	2.52923387106467
O	-3.43972066647743	0.09628398827104	3.37742321682694
H	-3.46862232173958	-0.85127933580078	3.19900825507913
H	0.08115648700053	-1.74395302975133	2.22742343042192
O	-0.39264553829445	-1.64510015672025	3.07268749346321
H	-0.39451235406801	-0.68096905531193	3.20437733547828
H	-4.39054377627969	-1.18406960736260	-2.22755174743623
O	-3.93723465374764	-1.96562621021536	-1.86838489792568

H	-3.35416435063313	-1.58847869766158	-1.18215490961039
O	2.00281039106079	0.53583130814610	-0.00508560755040
O	0.48096783147561	-0.21803143401885	-1.90172045478855
O	1.22236161303390	2.26367187972755	-1.67345445226808
P	2.22753183132558	-1.00315058435787	0.46559542800958
O	2.80448728202788	-0.68766024090402	1.92736900685736
O	0.86729946224687	-1.68841723043545	0.56361023229186
O	3.23562380938235	-1.69516247453729	-0.41185669901731
C	3.28427952425689	-1.80035096393339	2.72487920074187
H	3.67182957684865	-1.36579708050088	3.64472516710667
H	2.45976387587920	-2.47968830244259	2.95112600388296
H	4.08029938780729	-2.32665247167392	2.19344684834802
H	4.50371984434236	0.40528739005345	1.59089886192736
O	5.38697180632389	0.55713555844699	1.22522752677610
H	3.42857004313549	-0.97266079622456	-2.08616083367790
O	3.43900434704307	-0.44338291390884	-2.90556326067259
H	2.50228283909330	-0.27352427859679	-3.06764152286709
H	-0.25716547851164	3.42990549358090	-2.05302263966674
O	-1.13241736271178	3.83444287750939	-1.92783681053660
H	-1.70737793609587	3.05071121197776	-1.81591344920757
H	2.49202648199926	3.33124086494523	-0.83518432363552
O	3.33055915557643	3.69843212430630	-0.50670342154394
H	3.97275060294640	3.02253036506264	-0.79186284036506
O	-4.86601645681327	0.65861174536681	-2.44770720919773
H	-5.41882486747541	0.68134806691917	-0.56872542473210
H	-5.16916306289053	1.18413493832102	-3.19525636313368
Mg	-0.39769913145657	-1.95576995260998	-1.12395379856478
O	1.21665200327180	-3.12816642724631	-1.93403614623955
H	1.31853253040369	-2.99839494693739	-2.88469742773191
H	2.05616076415641	-2.82537485337297	-1.54441426150458
O	-1.41943378679517	-2.27215714309238	-2.95558975925575
H	-1.23951522760018	-3.12165150791688	-3.37433294505154
H	-2.38380942978312	-2.25470851881219	-2.77847484643027
O	-1.22523095267519	-3.71881421263360	-0.27292571515867
H	-1.83150340943289	-3.45359442813064	0.44603713938997
H	-0.51268594854685	-4.20728758065443	0.17911584045681
H	1.13999926988200	-3.49125440803192	1.02782757492025
O	1.10738706436503	-4.45922286179793	1.12614038125078
H	0.99282690873979	-4.60242927298549	2.07233106940527
H	4.38024123916801	0.95109652436782	-1.90468043957456
O	4.95912644433016	1.54659892845410	-1.39911424603656
H	5.08085373641108	1.11541735759508	-0.53312583639847
H	5.77015736379326	-0.32670451347698	1.17729694600783

Metaphosphate-Like Structure from Constrained Optimization, E -3394.520791309877

P	-2.31950465140087	0.88032465587683	-0.07977756917895
O	-0.21910782700916	1.46264754960382	0.22284471784034
O	-2.64980688269999	1.54880629871361	1.22745148290943

O	-1.90489731639993	-0.57896694456715	-0.10210981888099
O	-2.40030593554748	1.65789678661151	-1.36250341397357
P	0.85314822676548	1.18531494413853	-0.85522214090361
O	-4.51222502495561	0.20951495282352	-0.20264632914231
H	-4.15568987736201	0.56903705125777	-3.26850264106234
H	-5.02675284551770	0.89475731238343	0.24067568644219
H	-1.20914240591045	3.85304886153527	2.55781415913069
O	-1.74593444464456	3.30864534476220	3.15755712588915
H	-2.16057994885624	2.68007070571780	2.53780749303293
H	-1.97198251838443	-2.07767913243036	2.46878496240283
O	-2.77126726299366	-2.24113024092932	1.92900111739640
H	-2.66618995043783	-1.58888844857899	1.20948140254812
H	-0.34200341733613	1.86732008428415	3.25967112450750
O	0.26223805274183	1.16995004018573	2.95895888910455
H	0.16848957444440	1.20061265054667	1.98666140618004
H	-0.13250843426779	3.19964266360114	0.76926404017382
O	-0.15671031423510	4.16256364540475	0.93830414263144
H	-0.50467804342889	4.50487964529144	0.10234604334093
H	-3.58633469312894	0.66219637744525	2.51232359624101
O	-4.04486358441729	0.11095856962891	3.17488596427679
H	-3.78228478078125	-0.78726534627834	2.92236324388088
H	0.16115448647057	-1.72317420282162	2.29022059403114
O	-0.30617295463922	-1.59161597029261	3.13463623026972
H	-0.24093975359960	-0.62491844521940	3.24946027087220
H	-4.49730977895258	-1.69952150474327	-2.34868428920362
O	-4.10301760478881	-2.52611037329057	-2.01940237884146
H	-4.06080803232361	-2.41034710719259	-1.06242738134641
O	2.16999496966296	0.62299274057779	0.01765227575222
O	0.47352250941596	0.01703982711208	-1.77211865814089
O	1.36275228523377	2.42396059726251	-1.56094196971414
P	2.32006047438164	-0.91393301452838	0.46115421297069
O	2.98615517491015	-0.66776803069265	1.90593052757919
O	0.93415156285446	-1.54171153130145	0.62195613785975
O	3.24948250687599	-1.67389390504090	-0.45306781198710
C	3.39424399191727	-1.82655618027386	2.67283998415508
H	3.86730097588442	-1.44174935411950	3.57505335286681
H	2.52054795826605	-2.42617259639268	2.93744893561606
H	4.10918527790129	-2.42406669119110	2.10221311159765
H	4.72589726487588	0.24690787226421	1.46075427641949
O	5.58577894453689	0.39295542709043	1.03930576763031
H	3.30322002991611	-0.97675974958513	-2.20241038093180
O	3.11010443817159	-0.42452153191878	-2.98066027987953
H	2.17067549567391	-0.21607202258325	-2.84930987047576
H	-0.09430856016725	3.58812728328835	-1.89414522160642
O	-0.97577004523572	4.00208491292361	-1.92712488823747
H	-1.56416560493597	3.25577609401133	-1.70504628438659
H	2.81324520711361	3.28544205386797	-0.96107767166148
O	3.69612597368359	3.63385664565724	-0.72885712072230

H	4.28383554754595	2.91250293631904	-1.01618521892477
O	-4.89053351386750	0.04124404947653	-2.93254306543377
H	-4.78808645954938	0.23236157475603	-1.14118357304126
H	-5.66637773953445	0.32940217480542	-3.42691902050172
Mg	-0.41933791591337	-1.67835906220243	-0.99952708325012
O	1.10021516819984	-2.91441942183000	-1.92614012444941
H	1.20276913141770	-2.70220456498211	-2.86174772931818
H	1.96065323480276	-2.69616040163904	-1.52446219618890
O	-1.54557612855503	-1.93437737000294	-2.76284314287667
H	-1.19894422045939	-2.58864605950923	-3.37983238021439
H	-2.46330692904286	-2.21881746659548	-2.55085988411708
O	-1.24290664598551	-3.47433608140213	-0.17813173044253
H	-1.84503261150221	-3.25931654663243	0.55658046380862
H	-0.52530072482277	-3.98917650410993	0.23582781790564
H	1.19254355864795	-3.36411256865625	0.98439885074920
O	1.12720692459790	-4.33273154015992	1.06487386197247
H	1.06832654758934	-4.49245161050101	2.01355283348937
H	4.37439168687556	0.81922429672271	-2.04082514323759
O	5.09323941535622	1.31120441269382	-1.61175379335547
H	5.20133957594426	0.90415123285189	-0.73257258132079
H	5.96874621091530	-0.49053874929832	0.98648968347474

Mg·MeTP^{2-} Hydrolysis in Implicit Water with a Custom Dielectric of 20.4930

Custom Implicit Water Mg·MeTP^{2-} Hydrolysis Reactants, E -2170.602637576992

P	-1.42463651701038	0.15017868918596	-0.40978821985370
O	-0.02581479214449	1.03068571989776	-0.76638594415811
O	-1.70163188114141	0.52651442937067	1.03337268705469
O	-1.01811189273780	-1.33424021753472	-0.57540468336581
O	-2.44690082539195	0.59985633215908	-1.44378768976258
P	1.11346336887312	0.82572497564514	-1.86984660365923
O	-4.31581480898947	1.01172615903197	1.75944076074607
H	-4.08738162679587	0.98737505972777	-1.18942810041521
H	-3.37577217589995	0.83225786152862	1.54798865012734
O	2.42040185412476	0.45974628025572	-0.94473294840016
O	0.78847203796413	-0.42118445418907	-2.68961518297164
O	1.42133560903593	2.09696585293813	-2.58185456290785
P	2.64742186919824	-0.81234152034555	0.04544944710489
O	1.74717030693019	-0.33262609578754	1.29219391685841
O	2.00766623695677	-2.05103897576191	-0.58007750226228
O	4.10015767054620	-0.88695056017943	0.38331484282243
C	1.22637943184532	-1.31115242919226	2.21311733087730
H	0.69867366442776	-0.75292667671299	2.98542523794104
H	0.53184078682637	-1.97908381147685	1.70002841054261
H	2.04112116399999	-1.88442785537826	2.66466854917645
O	-5.03767952051263	1.19439209653126	-1.02138209076324
H	-4.71952624283372	1.11646673183104	0.88180764302079
H	-5.14773593836652	2.10518928154534	-1.31292048064019

Mg	0.37432222109541	-2.09786687308987	-1.69098346711201

Custom Implicit Water Mg·MeTP^{2-} Hydrolysis Transition State, E -2170.564365044551

P	-1.84270496312430	0.41462884429887	0.38674269507278
O	0.54640724930154	0.35012750113782	0.29792078982795
O	-1.80434572162658	0.68174774338274	1.85306877539884
O	-1.75123739391978	-1.02745044269092	-0.11028745753567
O	-1.90996526156607	1.51222774861284	-0.63123972934779
P	1.32688091879847	0.67671391215180	-1.01084523515881
O	-4.11490947202656	0.19497885099054	0.42609699897507
H	-3.34870440812595	1.58949958840796	-1.74873501845425
H	-4.43753521957431	0.77707133368144	1.12524306369557
O	2.85897532303471	0.07241176561542	-0.70853496249071
O	0.73872738427898	-0.29557148030244	-2.06390353594832
O	1.52187129742853	2.11555186118127	-1.38113144179656
P	3.21803159660843	-1.47470784996355	-0.36328329891143
O	3.92088658065617	-1.34879787761990	1.08235724551517
O	1.91050936369482	-2.25668175813128	-0.20869834179121
O	4.24241783419513	-1.99607555093379	-1.31863744861973
C	3.17734191330247	-0.78445874095794	2.18061125397021
H	3.81260317509384	-0.87567145175450	3.06127585048053
H	2.96133071912206	0.26854662960731	1.98506595667784
H	2.24670936425066	-1.33585056109406	2.33562404265338
O	-4.25567749745048	1.43759840871771	-2.08094967231304
H	-4.44057262236282	0.58715933828111	-0.40826146337993
H	-4.65727881066869	2.31219710534287	-2.11898813237549
Mg	0.08023965067973	-1.59519591796134	-0.58051093414441

Custom Implicit Water Mg·MeTP^{2-} Hydrolysis Products, E -2170.597781744798

P	-3.89114672614762	0.32682122602598	0.66248504042638
O	0.19111716996471	1.18802048397563	0.09552339589406
O	-5.04245781652383	0.16398050279580	1.64197088404701
O	-2.79113872231416	-0.76500232405738	0.82897045224272
O	-3.28737048206273	1.72203695909042	0.54757552903443
P	0.60841190922121	0.81944399272771	-1.32927028664591
O	-4.49056965124535	-0.06925584146074	-0.85312283173641
H	-1.35094989820725	0.14553196915264	-2.18807886347476
H	-5.36286765642394	0.31847748639137	-0.99615030716573
O	1.88983567659668	-0.18935947703418	-1.21539101862320
O	-0.52721886365935	-0.25956156712633	-1.76202652905980
O	0.89438199206224	1.86679479602334	-2.34434255191987
P	2.06764730241240	-1.36109350684400	-0.09007158146835
O	2.93945624689671	-0.52864905160819	0.98087188652705
O	0.69646799771913	-1.66581314448996	0.50974860525359
O	2.83225246820275	-2.49836713823396	-0.68210755001495
C	3.34594876132662	-1.20845612278433	2.18698508933437
H	3.91234549654236	-0.48501158924998	2.77200712941922
H	2.46845261489019	-1.53496536357371	2.75165803370351

H	3.97959591481791	-2.06560854521181	1.94400678208475
O	-2.67494760902577	0.77689022012603	-2.75722388313391
H	-3.37274378647177	0.49151894815968	-2.13050169402918
H	-2.59840788284284	1.72963157540502	-2.62585161822941
Mg	-0.91411445572821	-0.50340448819897	0.52359588753444

Mg·MeTP^{2-} Hydrolysis in Implicit Acetone

Implicit Acetone Mg·MeTP^{2-} Hydrolysis Reactants, E -2170.559884387964

P	-2.12916946532552	-0.65809220377956	-0.58281201409658
O	-0.77385693342004	0.36504722113354	-0.44335984574124
O	-2.72934291985563	-0.64033175989338	0.80426590236955
O	-1.49664297822453	-2.02859457133465	-0.95980067181882
O	-2.96966405666870	-0.07409946930442	-1.70720056936839
P	0.33204648766005	0.76531798559130	-1.53218146137941
O	-4.42332785952649	1.45376012834467	1.37474251010793
H	-3.79581710344562	1.34296269861201	-1.57232793594606
H	-3.81887791454879	0.69163751197716	1.24035333316482
O	1.70240085462194	0.66264276706278	-0.62324842053817
O	0.42465130277453	-0.37371943241092	-2.55503721104352
O	0.21228323786471	2.16291881861947	-2.01785506424936
P	2.38272310833524	-0.63352213180104	0.07763759074802
O	1.91226504473032	-0.36309398664571	1.60337682893134
O	1.67255815095368	-1.90021946484371	-0.41337628611403
O	3.86417450618378	-0.57949003535864	-0.06563315129549
C	2.34303515194128	-1.29840526276480	2.59978956491599
H	1.94503069793339	-0.94913800029007	3.55363113959492
H	1.95015270396295	-2.29862146777461	2.38911528069128
H	3.43598236305667	-1.33436007507567	2.65366198856309
O	-4.30245775647769	2.18948788681010	-1.43384282268369
H	-4.47551340527335	1.84272771060277	0.48804065472710
H	-3.70685554811406	2.88879449880890	-1.71985160565478
Mg	0.26164233086194	-2.16036936628549	-1.72748773388450

Implicit Acetone Mg·MeTP^{2-} Hydrolysis Transition State, E -2170.515342147185

P	-2.08515822215247	0.65196362740603	0.02040193927137
O	0.50137604351346	0.74563748990014	-0.07840828384693
O	-2.12103688203146	1.17647823979526	1.40648756145799
O	-1.80445210145935	-0.82903389494459	-0.20785600788160
O	-2.28888842227002	1.51058592639147	-1.18415468740671
P	1.42044522760993	0.73843503888677	-1.33635551270487
O	-4.46307568164455	0.20219064870331	0.12934416659910
H	-3.83076265793055	1.82371817723938	-1.93078543303025
H	-4.72214369156563	0.63262047361576	0.95266353547888
O	2.89421002229963	0.16460655074116	-0.73236918857617
O	0.89689431757565	-0.46130568015436	-2.17642441891280
O	1.73763422667060	2.02726951463561	-2.02129369520996
P	3.11769021350581	-1.20449384760515	0.08413602159417

O	3.48938197733759	-0.57194444618538	1.53884937547435
O	1.78087140585782	-1.95247585038259	0.23409798830225
O	4.26562652896065	-1.98914510147061	-0.46230909419764
C	3.89125808620848	-1.48294355468158	2.56464913551910
H	4.09075586897956	-0.88874970535257	3.45837830275999
H	3.09540858963633	-2.20366621319388	2.78396209919556
H	4.80115107384637	-2.01956418948557	2.27612532559990
O	-4.79272825070220	1.78040843113059	-2.12267230545150
H	-4.85036236379635	0.74186042241845	-0.58628675599092
H	-5.10514168204437	2.69134195471556	-2.12496760054103
Mg	0.08104637359511	-1.28379401212325	-0.48521246750228

Implicit Acetone Mg·MeTP^{2-} Hydrolysis Products, E -2170.525107081054

P	-3.28925012615970	1.30373265321781	-0.33963806078777
O	1.56444788972748	2.12569631466592	0.10092340024654
O	-4.20404630561423	1.74986243214889	0.74785819424758
O	-1.79862597167466	1.33710776962868	-0.02816363263187
O	-3.65420921105309	1.46653554800835	-1.78981800921622
P	2.42363138421653	1.61287220381422	-1.09025674066716
O	-3.54413540121221	-0.61714396342869	-0.22811552242983
H	-4.47501884033561	0.10156628429959	-2.55173906696711
H	-4.02162666919669	-0.85962650150195	0.58063281564237
O	3.07470261292649	0.15678264802650	-0.53158592717339
O	1.35924066903809	1.15295577928498	-2.13172153637397
O	3.58731262237432	2.42974902511717	-1.55203905800089
P	2.22849513465909	-1.07374577913080	0.07619624305625
O	2.74482552943087	-0.99244386060929	1.62188150002286
O	0.73144532449956	-0.73301942076781	0.07129844998339
O	2.63261236104731	-2.37097101918088	-0.54671395678416
C	2.24464721205556	-1.98256174391869	2.52327784287289
H	2.69863933766062	-1.78810153387165	3.49681265596419
H	1.15492465153716	-1.91344450831518	2.61519377043861
H	2.51985620077063	-2.98966488900266	2.19168435001163
O	-4.71169599410762	-0.85341482747810	-2.51439558097209
H	-4.07709805897041	-0.91752186863833	-1.02821257741129
H	-5.66769434345454	-0.91110224181128	-2.62872777778847
Mg	0.00059999183508	0.98050149944321	-0.59337177528211

Re-Optimization of the Implicit Acetone Transition State in Custom Implicit Acetone with an Abraham's Hydrogen Bond Basicity (solb) of 0.90

Re-Optimized Implicit Acetone Mg·MeTP^{2-} Hydrolysis Transition State with solb 0.90, E -2170.521169918546

P	-2.09206615863866	0.64867346915013	0.00941414406206
O	0.50118781981652	0.74808720518579	-0.08242777177729
O	-2.13638964498093	1.18027713607894	1.39248397005640
O	-1.80525178468057	-0.83234109088249	-0.20936007973875
O	-2.29805340530800	1.49942226346921	-1.20012195656402

P	1.42496518668037	0.73242592286799	-1.33716425752165
O	-4.48459202330736	0.19200298219979	0.11360381722708
H	-3.84468092652964	1.83799739997475	-1.93049856142838
H	-4.73943149414813	0.61448124271196	0.94225182490139
O	2.89687376272027	0.16281517497179	-0.72478999168088
O	0.90394338892709	-0.47259672031185	-2.17142795122875
O	1.74332445925898	2.01729271883106	-2.02924945639168
P	3.12035239252129	-1.20627128688880	0.09233293902377
O	3.50139947314070	-0.57265130051227	1.54438877462586
O	1.78176147219227	-1.94982323408872	0.24969087108279
O	4.26301840032088	-1.99560056604217	-0.45870298530056
C	3.91096206879150	-1.48334780417219	2.56755961479358
H	4.11105834609308	-0.88953541095938	3.46148549814808
H	3.11938599019767	-2.20800811522626	2.78943777639051
H	4.82235917564039	-2.01546294946827	2.27552245215886
O	-4.80756835753828	1.80786927822761	-2.11890082456443
H	-4.86680566318997	0.74524785081859	-0.59391787667573
H	-5.10938518423069	2.72228303171338	-2.10565583607660
Mg	0.08363270625129	-1.28323719764869	-0.47595413352166

Attempts to Optimize the End States of $Mg·MeTP^{2-}$ Hydrolysis Within an Explicit Water Shell in Implicit Water

Aqueous $Mg·MeTP^{2-}$ Hydrolysis Attempted End State: α-β-γ Mode, E -3394.527205059928

P	-3.11454601572664	0.86894111860033	-0.03833253500671
O	0.28284079509027	1.72676351995196	0.72892954688317
O	-3.72790009943987	1.26589780343723	1.30478593430756
O	-2.26574880130999	-0.42071722848416	0.04902508056486
O	-2.49258930773831	2.00278031974424	-0.83058176371914
P	0.99908301436437	1.39529453942998	-0.57344209192327
O	-4.41855139001528	0.34044703536185	-0.97954908764588
H	-2.74733502082468	-0.54485568529593	-3.12843953808616
H	-5.18506218297534	0.92108377663431	-0.88145290705564
H	-1.87906812484123	3.57745084128014	2.32168479864543
O	-2.44463901546595	3.05908753011623	2.92202223351394
H	-2.91817696117151	2.43390628121631	2.33138952202142
H	-1.73353245885840	-1.87626649653304	2.77563457260999
O	-2.56349872523780	-2.02435872769119	2.28012639628800
H	-2.53534946799202	-1.33861674788792	1.58105826018603
H	-1.00781128237588	1.86451389887091	3.39909810310136
O	-0.19162732706429	1.33289478702935	3.37614914528763
H	0.07770212210028	1.38909954517670	2.43587294853127
H	-0.15529607574954	3.37920867565113	1.05262631335000
O	-0.51513793504371	4.28383207855958	1.19117090459778
H	-0.78148199305630	4.53948620130573	0.29534937904948
H	-4.67613328747488	0.05832166954052	2.18893242521566
O	-5.09538257720465	-0.67396025378851	2.68887218746160
H	-4.36568260390561	-1.30592783247823	2.77560248251865

H	0.38889107346195	-1.49390292998318	2.45778724762411
O	-0.02482796119241	-1.45870749612283	3.33956258289120
H	-0.14665186169265	-0.50059521250507	3.48741360774304
H	-4.71803484074209	-2.01928966378361	-1.37006125812755
O	-3.99436509953294	-2.56849291921035	-1.04504277561365
H	-3.53440663217845	-1.97740319197580	-0.42607774070543
O	2.41107295841333	0.60195471344196	-0.09664936249799
O	0.28464981351169	0.34957992389168	-1.45530848603553
O	1.49479268063878	2.60020256206707	-1.35992418561389
P	2.40986279022351	-0.93149294696111	0.36090436426369
O	3.29536267724846	-0.78393669285553	1.69984276198797
O	0.98814012943565	-1.37880547205050	0.71407718453352
O	3.10667318462604	-1.80358953894560	-0.65812797913197
C	3.66134929436320	-1.98550586561265	2.41937236494727
H	4.34598821336178	-1.67245731144660	3.20634143868974
H	2.77156078195429	-2.44057518201890	2.86000774432120
H	4.15965569268654	-2.69063022438226	1.74989479815883
H	5.04649322399119	-0.05257872162052	0.99116110816187
O	5.84013326296583	0.02359930670038	0.44121000973090
H	2.91446264990481	-0.99853132885010	-2.41281603414152
O	2.65645753584637	-0.37722543311925	-3.11483573071521
H	1.79557813203368	-0.05637755220229	-2.79979452018768
H	-0.03200118868971	3.67116188723711	-1.69658404001999
O	-0.91878655703519	4.07374730699298	-1.74211586253865
H	-1.50791190627161	3.35596007249568	-1.43777987658584
H	3.15103868548011	3.19891255530404	-1.15049624492522
O	4.09872144098984	3.44167200627188	-1.11770177283023
H	4.53648915532606	2.64961320191704	-1.47691844143741
O	-3.47455606699336	0.14159563978999	-3.33469880312927
H	-3.95715147068351	0.28345430390881	-2.43898455006853
H	-3.04435155447024	0.97752988608883	-3.57835132429712
Mg	-0.60132316543952	-1.34923629074032	-0.75046705466199
O	0.73527534127013	-2.64975185319158	-1.89882141472354
H	0.82567999735699	-2.34577950829808	-2.80996489084182
H	1.63764143569592	-2.58265817659283	-1.53209867325652
O	-1.88410544801529	-1.70527058111131	-2.54844496528437
H	-1.49108653798709	-2.23954056115552	-3.25067190842572
H	-2.62618720182648	-2.23609058235286	-2.17528305249512
O	-1.30253142554370	-3.18999603615616	0.06542742815456
H	-1.81332769563570	-3.00356603477502	0.87654420936477
H	-0.55713644075148	-3.74101549907744	0.36578903029606
H	1.17415356834553	-3.21984893496004	1.07796305882745
O	1.10609605533903	-4.19104407944142	1.10201415622093
H	1.06942398637253	-4.40861789814228	2.04026310369672
H	4.24996453087960	0.59826368997689	-2.44092903788414
O	5.09555481385559	0.97283871790679	-2.14439642497555
H	5.27248124274970	0.56409949392335	-1.27725621342016
H	6.14895342827036	-0.88585819802091	0.35573411426077

Aqueous Mg·MeTP^{2-} Hydrolysis Attempted End State: β-γ Mode, E -3394.543277623522

P	-2.76000562409478	-0.85555323582692	0.11854510264510
O	0.43673864247887	-1.14736194624250	0.53844733345309
O	-2.33058187658528	-2.27793844607994	-0.12267931998081
O	-2.12268821767278	0.25772800983270	-0.69043314609413
O	-2.65630226598894	-0.56823507653670	1.69507087603985
P	1.06015788335060	0.13320979824582	1.07303519084451
O	-4.34885299105746	-0.69080268413159	-0.23746083805171
H	-3.19666418374736	0.18363337835319	2.04584333322102
H	-4.90001550625374	-1.39718899304040	0.12631139438614
H	0.61750182108015	-4.00967180479574	-0.15814082550106
O	-0.25883875373765	-4.25412247818144	-0.50705169181168
H	-0.86636465293851	-3.55447066530142	-0.21418463135578
H	-3.47765806543111	-0.84326342938632	-3.40762832758912
O	-4.42572443723484	-0.97111432700285	-3.23238630812754
H	-4.52709996239592	-0.71437306820221	-2.30426515056831
H	0.23875581352042	-2.84849014673359	-1.94626874646483
O	0.46420738769972	-1.91872259184656	-2.10031110154442
H	0.42533899521870	-1.53546104635841	-1.19368611984305
H	0.15137893455059	-2.44537317264268	1.75892851465979
O	-0.03332445707737	-3.05265041745248	2.50491962669127
H	-0.26387400149469	-2.44712508566283	3.22256661921968
H	-3.16730981537060	-3.20538838665719	-1.45065769202401
O	-3.65753795992320	-3.61494044795652	-2.18850722399725
H	-4.02385423053739	-2.84941146441331	-2.65838234670720
H	-1.84890048904657	-0.11173519744361	-2.43651742846257
O	-1.57901092038589	-0.44773789844558	-3.31357502103707
H	-0.92984347322948	-1.13474587704446	-3.07083575012480
H	-5.02521171904928	1.83694089390260	1.26799388302476
O	-5.04537966285035	2.04906016708994	0.31988487986186
H	-4.93640633307203	1.18026104862279	-0.09690933935079
O	2.29744612433107	0.41307641091199	-0.01581051876923
O	0.16019906605715	1.37621995183659	0.92987496511739
O	1.66959082093201	0.02939586654559	2.46788998841655
P	3.83931273259541	0.86058842367544	0.12664720649608
O	4.17927511781952	0.66584051816114	-1.45958747020639
O	3.94580469514620	2.31958330691615	0.50550253171547
O	4.63970347961765	-0.13122192581948	0.93616317158852
C	5.54267544520701	0.87417509128555	-1.87670718543176
H	5.56531573378258	0.71730146981931	-2.95483708214813
H	5.85928643608953	1.89547014536400	-1.64735159651163
H	6.20487333914491	0.15597092000781	-1.38569896860025
H	1.23369610194002	0.89755386739173	-1.66318500453035
O	0.46205552013208	1.32955682165904	-2.06002433803402
H	0.08729202471303	0.69864185130904	-2.69157503555706
H	2.52014465520059	3.25219978498702	1.05053884337195
O	1.67069343428181	3.63321885374507	1.35381282104540

H	1.09153942564601	2.84577807605274	1.40368379600983
H	0.15300840103319	-0.27361883037143	3.55247341620744
O	-0.70206943294284	-0.51310064823346	3.95856963551458
H	-1.32289613073920	-0.51418478125269	3.21515464566065
H	3.96130728569818	-1.56267815701072	1.81050781383686
O	3.38082196063768	-2.15626093677478	2.32682153580859
H	2.65529852964436	-1.54250148973802	2.55277297156263
H	2.74462229360419	-3.19746620019056	0.92512484898060
O	2.51367099427201	-3.70610967831622	0.12308191889018
H	2.82482517227620	-3.14583427137084	-0.60785196216228
H	3.58246461282397	-1.12289514407471	-1.94970436421400
O	3.26278599078902	-2.01261547019422	-2.16689846701049
H	2.29403366231339	-1.91453430329226	-2.24602365328768
O	-4.13948883514076	1.31910214155863	2.90796757235535
H	-4.54691608609296	0.94783363871172	3.69962637629778
H	-3.58707621662681	2.04621025945570	3.22059377473142
Mg	-1.00098405309949	1.96823337260106	-0.61662668512591
O	-0.05385003423057	3.89157905708844	-0.77951274419021
H	-0.64260802573187	4.64892776824921	-0.68819886877284
H	0.62626565700120	3.99137318327325	-0.07959289854037
O	-2.41674810045283	2.87292685620799	0.67040049705128
H	-2.39275118353805	3.83573478312212	0.70074095320139
H	-3.34794598766445	2.63269237412695	0.47668453517554
O	-2.21886031766011	2.59372968205978	-2.29548959780198
H	-2.99507657125300	2.01833388194890	-2.31874376023256
H	-1.78328761628070	2.45405806990576	-3.14561936331924

References

(1) Rappe, A. K.; Casewit, C. J.; Colwell, K. S.; Goddard, W. A.; Skiff, W. M. UFF, a Full Periodic Table Force Field for Molecular Mechanics and Molecular Dynamics Simulations. *J. Am. Chem. Soc.* **1992**, *114* (25), 10024–10035. https://doi.org/10.1021/ja00051a040.

(2) Rosing, J.; Slater, E. C. The Value of ΔG° for the Hydrolysis of ATP. *Biochim. Biophys. Acta BBA - Bioenerg.* **1972**, *267* (2), 275–290. https://doi.org/10.1016/0005-2728(72)90116-8.

(3) Wang, C.; Huang, W.; Liao, J.-L. QM/MM Investigation of ATP Hydrolysis in Aqueous Solution. *J. Phys. Chem. B* **2015**, *119* (9), 3720–3726. https://doi.org/10.1021/jp512960e.

(4) Takahashi, H.; Umino, S.; Miki, Y.; Ishizuka, R.; Maeda, S.; Morita, A.; Suzuki, M.; Matubayasi, N. Drastic Compensation of Electronic and Solvation Effects on ATP Hydrolysis Revealed through Large-Scale QM/MM Simulations Combined with a Theory of Solutions. *J. Phys. Chem. B* **2017**, *121* (10), 2279–2287. https://doi.org/10.1021/acs.jpcb.7b00637.
